# Development of a High-Resolution Tandem Mass Spectral Library for Pyrrolizidine Alkaloids (PASL)

**DOI:** 10.1038/s41597-025-05940-7

**Published:** 2025-10-20

**Authors:** Leonie V. Straub, Patrick P. J. Mulder, Han Zuilhof, Federico Padilla Gonzalez, Laura Righetti

**Affiliations:** 1 Wageningen Food Safety Research, Wageningen University & Research, Wageningen, The Netherlands; 2 Laboratory of Organic Chemistry, Wageningen University & Research, Wageningen, The Netherlands; 3 College of Biological and Chemical Engineering, Jiaxing University, Jiaxing, 314001 China

**Keywords:** Mass spectrometry, Secondary metabolism

## Abstract

This Data Descriptor reports the submission of a High-Resolution Orbitrap Mass Spectral Library of Pyrrolizidine Alkaloids (PASL) to public repositories. The library contains 165 tandem mass spectra (MS/MS) from 84 pyrrolizidine alkaloid (PA) standards, along with 18 additional PAs manually annotated in crude plant extracts. This collection comprises most commercially available PAs and a unique selection of annotated (in crude extracts) and synthesized compounds. The PASL serves as a valuable resource for identifying PAs in complex mixtures without analytical standards and facilitates the annotation of novel PAs through molecular networking approach. To ensure high quality, the library was validated by dereplicating its spectra against the GNPS libraries and applying it to the annotation of two PA-producing plant species. The spectra of the PASL can be accessed through GNPS (https://gnps.ucsd.edu/ProteoSAFe/gnpslibrary.jsp?library=PYRROLIZIDINE-ALKALOID-SPECTRAL-LIBRARY), with accession numbers ranging from CCMSLIB00014205782 to CCMSLIB00014205946. The PASL in .mgf format can be downloaded from GNPS under: https://external.gnps2.org/gnpslibrary. The PASL enables rapid and straightforward annotation of both known and novel PAs, accelerating research in food safety and related fields.

## Background & Summary

Natural toxins are a global concern for food safety. Plant toxins, in particular, pose a risk to humans, increased by diverse factors such as a rise in the popularity of plant-based diets, globalization and climate change^1–3^. One important class of plant toxins are pyrrolizidine alkaloids (PAs), a group of specialized metabolites suspected to be produced by up to 6000 species of the *Boraginaceae*, *Asteraceae* and *Fabaceae* families^4,5^. Their significance for food safety is historically proven, as over the last 120 years multiple incidents concerning both humans and livestock have been associated with PA intoxication^6–8^. In 2021, the European Union passed (EU) No. 2020/2040 as the first regulation to control the levels of 35 PAs in different food commodities, which since was overtaken in 2023 by regulation 2023/915^9,10^. This resulted in more than 150 notifications registered between 2021 and 2024 in the European Rapid Alert System for Food and Feed (RASFF). Most notifications concerned the import of seemingly harmless dried herbs such as oregano and cumin. Human poisoning with PAs is typically not caused by the intentional consumption of PA-producing plants, but by ingestion of plant-based products or phyto-therapeutics contaminated with PA-rich weeds^11–13^. After the human consumption of contaminated products, PAs exhibit hepatotoxicity in cases of acute poisoning, and carcinogenic or genotoxic properties after chronic exposure^14,15^. Particularly 1,2-unsaturated PAs are associated with higher toxicity, as they form DNA and protein adducts after metabolism in the liver^16^. These toxic effects and their wide distribution in the plant kingdom increase the relevance of PAs in the field of food safety, especially for children or individuals with pre-existing conditions^17–19^.

**Fig. 1:**
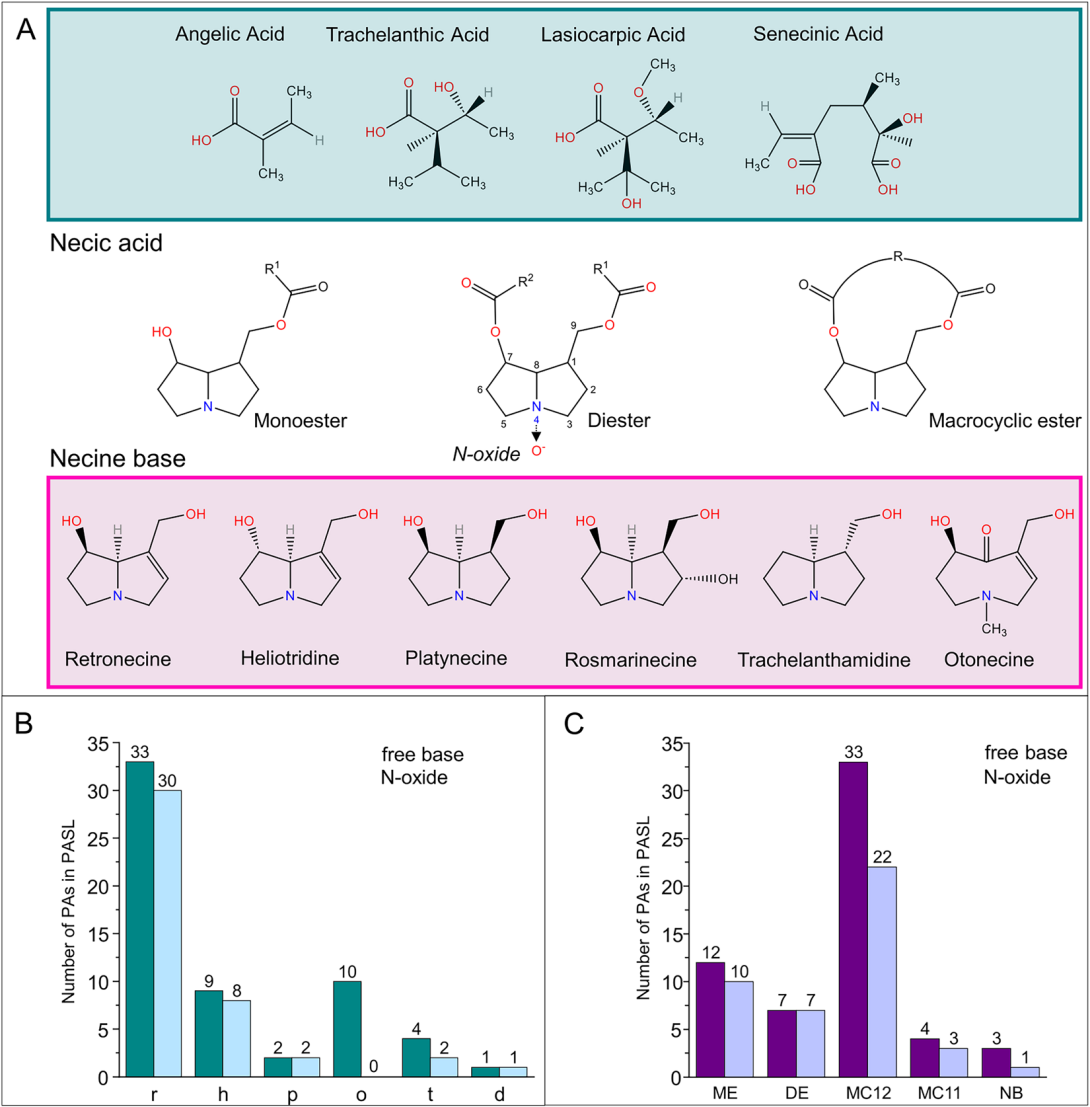
Chemical structure of PAs (**A**), collection of PAs (N = 102) commercial standards, in-house synthesized compounds, and annotated from crude extracts classified based on their necine bases (**B**) and their class (**C**). Abbreviations: retronecine (r), heliotridine (h), platyphylline (p), otonecine (o), trachelanthamidine (t), rosmarinecine (d), monoester (ME), diester (DE), macrocyclic esters with 12-membered ring (MC12), macrocyclic esters with 11 membered ring (MC11) and necine bases (NB).

As PAs play an important role in food safety, it is crucial to have a rapid overview of known and potentially novel PAs occurring in diverse food matrices. Chemically, the structure of PAs can be described as a necine-base core esterified with one or two necic acids, resulting in mono-, macrocyclic or di-esters (Fig. 1A). While there are four main necine bases, a huge variety of necic acids are known. Additionally, PAs can not only be found as free bases, but also as their corresponding *N*-oxides. This results in a huge variety of potential compounds that makes structural characterization challenging^20^. Due to their stereochemistry and numerous epimers, the correct identification and nomenclature of the molecule can be further complicated. Many of the available structures on websites such as PubChem are either incomplete and show unresolved stereochemistry in one or more stereocenters or show an incorrect stereochemistry. Additionally, the conversion from a correct structure to the isomeric SMILES is usually not reversible, as the structure retrieved from the isomeric SMILES is sometimes incorrect (S1). Therefore, in the supplementary section of this paper a list of correct structures and isomeric SMILES is provided for all compounds included in the PASL, based on the Joint FAO/WHO Expert Committee on Food Additives (JECFA) nomenclature (S2).

For the detection of PAs, two main approaches have been used: targeted and non-targeted analysis^21–23^. Targeted analysis focusses only on known or somewhat expected PAs. These expected, yet still unidentified, PAs can be investigated in a targeted manner based on the potential PAs chemical formula created by combinations of the necine base and necic acid(s) (Fig. 1). However, in this approach novel, unexpected or modified PAs can be overlooked. The alternative approach of a non-targeted analysis takes into account all compounds present in the plant sample. To annotate specifically PAs, prioritization strategies need to be applied to distinguish between possible PAs and other plant metabolites. An option to make this distinction is the use of computational tools such as molecular networking. In this approach, the compounds detected in a sample are grouped by the similarity of their MS2 spectra^24^. In 2023, Xu *et al*. applied molecular networking on PA-producing plants^25^. However, they criticized a lack of reference spectra in open-access libraries. This is a common issue of non-targeted analysis, as only a limited number of reference standards are commercially available. Additionally, the purchase of commercially available but costly non-regulated standards is often not feasible.

**Fig. 2:**
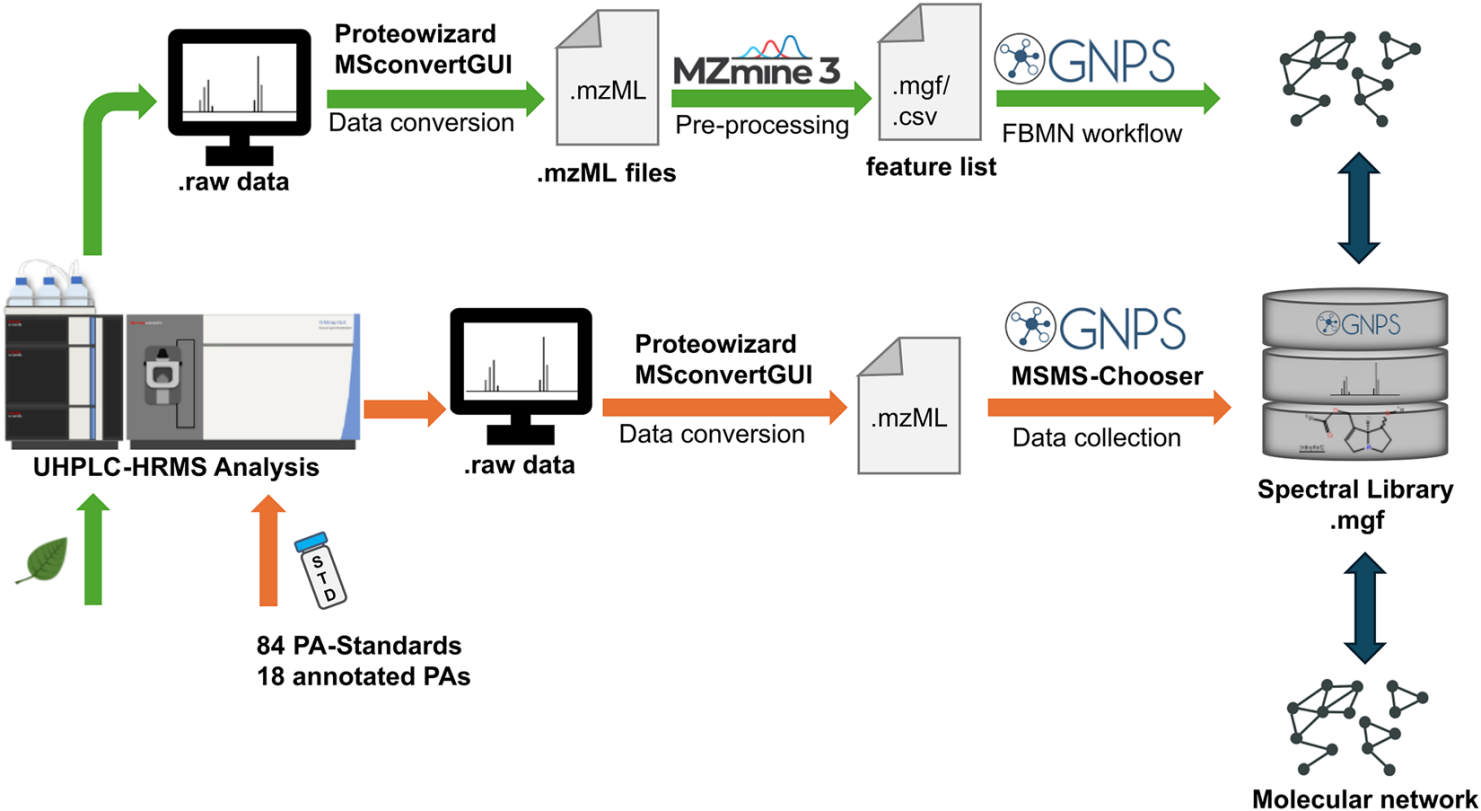
Workflow for the construction of the High-Resolution Orbitrap Mass Spectral Library of Pyrrolizidine Alkaloids PASL (orange arrows), and its validation by using classical MN (dark blue arrows) and FBMN of plant toxins (green arrows).

To enhance the availability of reference spectra, this study developed a unique spectral library for PAs—the PAs Spectral Library (PASL). This open-access resource provides the scientific community with high-quality spectral data for 102 PAs. Currently, only circa 80 PAs are sold commercially, which accounts for about 15% of the estimated number of existing PAs^26^. In the current study, we included 79 commercial standards, 5 in-house synthesized compounds, and 18 PAs annotated from crude extracts. This collection of standards represents the five different structural families of PAs, making them ideal to build a spectral library freely accessible to the scientific community (Fig. 1B,C). To ensure the high quality of the PASL, it was validated by using classic molecular networking (MN) to represent the structural diversity of the PASL, and feature-based molecular networking (FBMN) on plant samples to assess its application in the annotation of PA-producing plant extracts. (Fig. 2). Molecular networking allows the investigation of relationships between unknown and known compounds and therefore facilitates conclusions about the putative structure of novel molecules. The PASL is available at the Global Natural Product Social Network (GNPS)^24^. This web-based tool fulfils two main uses: it offers diverse computational tools such as MN or FBMN, but also gives a space to upload both research data and spectral libraries. By uploading the PASL to the GNPS repository, we expect to streamline the annotation of PAs in different food and non-food matrices, which in the long-run will help to improve food safety.

## Methods

### Sample preparation

Each PA analytical standard was diluted to a concentration of 500 μg/L with 10% UHPLC-grade methanol (MeOH) in ultra-pure water. Additionally, 18 PAs were annotated from 13 crude plant extracts. For each plant species, 10 mg freeze-dried and finely ground early stem leaves (harvested at 12 weeks) were extracted with 1 mL of 0.2% formic acid. Heliotrine was used as internal standard in the *Jaocobaea* plant extracts. All extracts were filtered through 0.45 µm filter vials and analyzed without additional dilution. The same extraction procedure was performed for the leaves of the *Jacobaea gnaphalioides* and *Heliotropium europaeum* samples that were used to validate the database.

### Data acquisition

The analysis was conducted on a Thermo-Fisher LC-HRMS system, equipped with a Vanquish Horizon UHPLC coupled to a Thermo Scientific Orbitrap IQ-X Tribrid mass spectrometer. For the chromatographic separation, a Waters Acquity UPLC BEH C18 (1.7 µm particles; 2.1 × 150 mm column dimensions) column was used. The flow rate was 0.4 mL/min and the temperature of the column oven was set to 50 °C. As aqueous mobile phase (A) 10 mM ammonium carbonate buffer (pH 9), as organic mobile phase (B) 100% ACN was used. The gradient started with 0% B, then increased in 0.1 min to 5% B and in 3 minutes to 10% B. The portion of B increased further to 24% at 7 minutes and 30% at 9 minutes. After 12.3 minutes the maximum of 80% B was reached and B was kept for 3 minutes at 80% till 16.3 minutes, after it was decreased to 0% at 16.5 min and then was kept there till the end of the run at 18.5 minutes. The injection volume of all standards and samples was 5 µL. For electrospray ionization (ESI), the Ion Transfer Tube temperature was set to 325 °C, the vaporizer to 350 °C, the source voltage to 3500 V and the sheath gas flow to 50 L/min. All samples were acquired in positive acquisition mode (ESI positive). The first experiment was a MasterScan of MS1 with an Orbitrap resolution of 120,000 FWHM and a scan range from 150–1500 *m/z*. The maximum injection time was 246 ms, the AGC target 400000. The minimum intensity was set to 50000 with a minimum relative intensity threshold of 20. The dynamic exclusion was after n = 1, with a duration of 2.5 s. The data-dependent acquisition (DDA) mode was set to cycle time (0.6 s). The second experiment consisted of a DDA scan, with a minimum of 9 data points and an Orbitrap resolution of 15000 FWHM in centroid mode. The “assisted collision energy” feature was with 15, 30 and 45 eV was used. Hereby hidden ion trap scans are performed to simulate the breakdown of the precursor, and the instruments picks the ideal collision energy for the MS2 scan.

### Database constitution

First, the data files were converted from.raw (Thermo) to the open file format.mzML in Proteowizard MSConvertGUI using settings described in S3. For the extraction of the MS2 spectrum of each PA, the MSMSChooser workflow (https://gnps.ucsd.edu/ProteoSAFe/index.jsp?params=%7B%22workflow%22:%22MSMS-CHOOSER%22%7D) was applied with the isomeric SMILES of the correct structure. The resulting table listed all possible adducts ([M + H]^+^, [M + K]^+^, [M + Na]^+^, [2 M + H]^+^) with corresponding precursor masses, SMILESs, InChIs, instrument information, origin information and data of the scientists involved. The generated results were carefully inspected and, if needed, manually corrected. Specifically, the scan number, precursor mass and intensity of MS2 spectra were checked. To ensure the high quality of the MS2 spectra, a minimum intensity of 1E5 was set for inclusion in the PASL. This resulted in a final table of 165 spectra including 102 compounds as protonated molecules, 20 as [M + Na]^+^, 2 as [M + K]^+^, and 41 as [2M + H]^+^. After the format of the resulting.csv table was validated, the Batch Validator workflow on GNPS (https://gnps-quickstart.ucsd.edu/validatebatch) was launched and the resulting job submitted to GNPS for publication. The final batch table submitted to GNPS can be found in the metadata on MassIVE as “PASL_batchfile_V02.tsv”^27^.

### FBMN on plant samples

The converted.mzML files were pre-processed in MZmine 3.9.0 (settings applied are displayed in S5) and the output uploaded to GNPS. In the FBMN workflow (CCMS ProteoSAFe Workflow Input Form), the precursor ion mass tolerance was set to 0.02 Da and the MS/MS fragment ion tolerance to 0.02 Da. A molecular network was then created where edges were filtered to have a cosine score above 0.8 and more than 6 matched peaks. All matches kept between network spectra and library spectra were required to have a score above 0.7 and at least 6 matched peaks (*Heliotropium europaeum*, matching PASL https://gnps.ucsd.edu/ProteoSAFe/status.jsp?task=d99d0ef6b5404522bee4c1f266c139e7, matching with GNPS libraries https://gnps.ucsd.edu/ProteoSAFe/status.jsp?task=95876d6a82734ec3af68d816c377860a; *Jacobaea gnaphalioides* matching PASL https://gnps.ucsd.edu/ProteoSAFe/status.jsp?task=462d8d50319243fcae583050b0fe4cbe, matching GNPS libraries https://gnps.ucsd.edu/ProteoSAFe/status.jsp?task=95876d6a82734ec3af68d816c377860a). The networks were visualized and modified with Cytoscape (Version 3.10.2).

## Data Records

All spectra mentioned in this descriptor were uploaded on GNPS. Each spectrum of the 102 compounds and their different adducts in the PASL has its own accession number from CCMSLIB00014205782 to CCMSLIB00014205946 on GNPS. The dataset (metadata files, raw files, .mzML spectral files, MZmine output and GNPS library matching files) is available on the Mass Spectrometry Interactive Virtual Environment (MassIVE, https://massive.ucsd.edu/ProteoSAFe/dataset.jsp?task=3b4a3d51da7b44e497b61d88a474fe33)^27^.

### Metadata

The MS2 spectra records in the MassIVE repository of the PASL library contain the following selection of additional information that is provided as metadata: a comprehensive overview of all PAs in the database with the name of compounds, molecular formula, adduct, scan number, precursor *m/*z, retention time, PA necine base, PA class, isomeric SMILES, InChI, InChI-Key, CAS-number (if available), Origin, Genus/species and GNPS spectra quality, a text file with information about the UHPLCHRMS method (“UHPLCHRMS_method_PAs.txt”) and a description of all the provided data (“readme.txt”).

## Technical Validation

### Validation of compounds

Most compounds implemented in the PASL were commercially available as pure analytical standards to ensure high quality (N = 79). Their structural identity and purity were determined by the producer. Five standards were either purified or synthesized in-house. These standards are categorized as “isolated” in the MSMSChooser table, as the workflow does not provide the possibility to categorize them as “synthesized”. Their structure was previously determined by using analytical standards (comply with Schymanski confidence level 2)^28^. The identity of the 18 additional PAs annotated from different *Jacobaea* species was previously reported by Chen *et al*.^29^. The same samples were used for the PASL, as no evident degradation in the compounds in the extracts was observed when comparing the MS2 spectra. The obtained mass spectra were individually inspected to verify the occurrence of different molecular ions (e.g. protonated, sodiated).

### Strategy for the validation of the PASL

The PASL was validated in three steps: (I) construction of a molecular network based on the spectra files of the PASLs using the PASL as reference library to explore the structural diversity and clustering patterns of PAs based on their chemical subclasses, (II) the dereplication and manual review of the spectra in the PASL against the PAs that were previously available on the GNPS library to highlight the relevance of this spectral library, and (III) the dereplication of two extracts of the early stem leaves of *Jacobaea gnaphalioides* and *Heliotropium europeaum* to demonstrate the functionality of the PASL.

#### Classical molecular network of PA standards

**Fig. 3:**
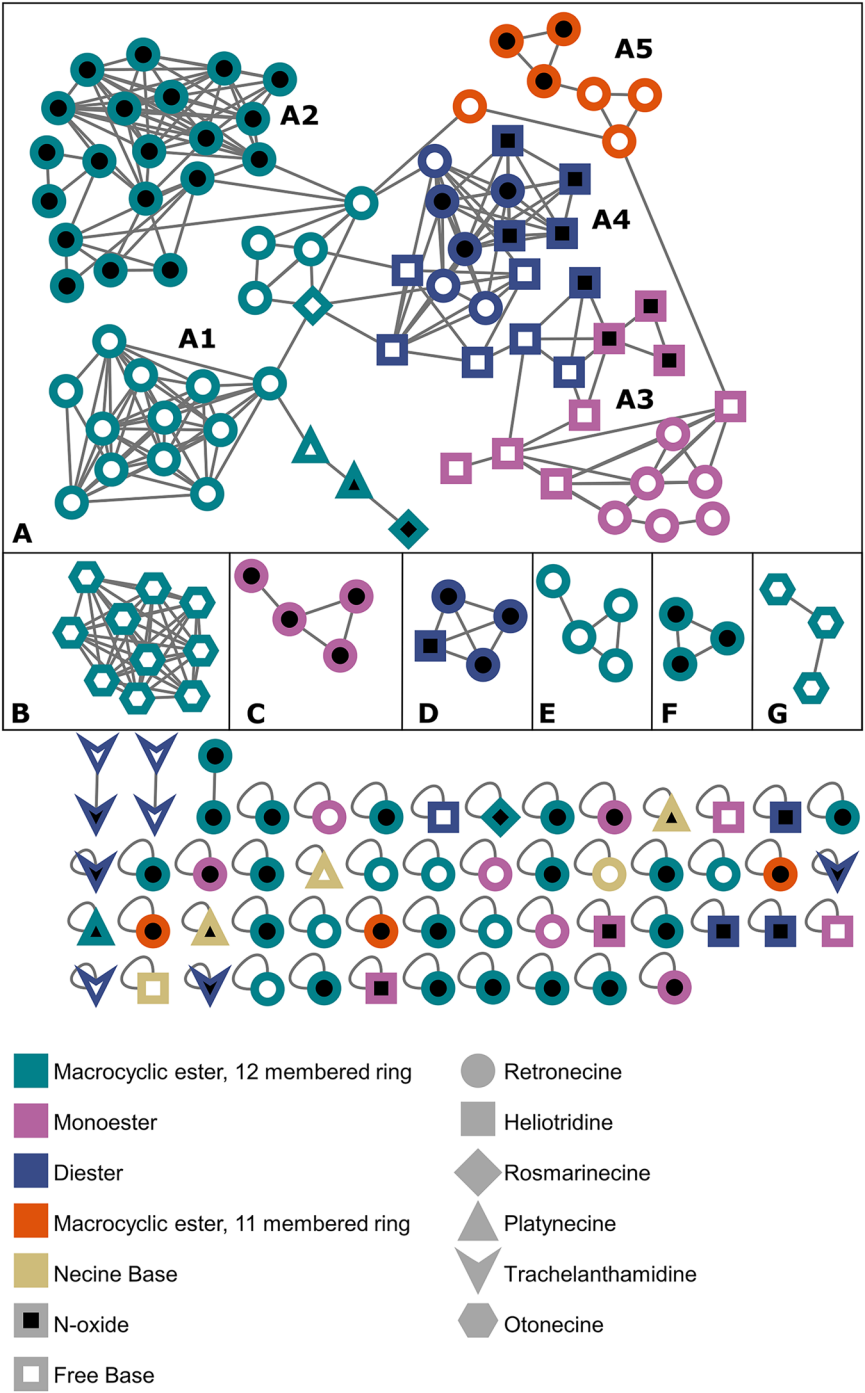
Classic molecular network, using color to represent PAs structural class, shape for the necine base, and fill color to indicate the PAs state (free base or N-oxide).

A classical molecular network was created using the online workflow (https://ccms-ucsd.github.io/GNPSDocumentation/) on the GNPS website. The precursor ion mass tolerance was set to 0.02 Da and a MS/MS fragment ion tolerance of 0.02 Da. The edges of the MN were filtered to have a modified cosine score above 0.7 and more than 6 matched peaks. The spectra in the network were then searched against the PASL. The MN was visualized using Cytoscape (Version 3.10.2). The nodes are colored according to their structural class, shaped according to their necine base and filled according to their state (free base or *N*-oxide). The MN consists of one big cluster (A), multiple smaller clusters (B-G), two pairs and single nodes. The clustering of PAs is based on their chemical class, state (oxidized or free base) and the necine base. cluster A can be roughly separated in five sub-groups: MC12 with their 12-membered macrocylic diester moiety (Fig. 3; A1, teal) and their *N*-oxides (A2, teal), mono-esters ME and their *N*-oxides (A3, pink), di-esters DE and their *N*-oxides (A4, blue) and MC11, with their 11-membered macrocyclic diester moiety, and their *N*-oxides (A5, orange). In the big A cluster, all PAs with the exception of platyphylline N-oxide and rosmarinine contain heliotridine or retronecine as their necine base, which is due to them being epimers and not being distinguishable by only using MS. Clusters B and G consist exclusively of MC12 with an otonecine base as [M + H]^+^ (B) and [2 M + H]^+^ (G) adducts. As otonecine is the only macrocyclic necine base, the separation of these clusters to all other compounds was expected. The clusters C, D and F comprise the *N*-oxides of DE, ME and MC12, respectively, and cluster E is composed of the free base MC12 which are not linked to the groups in cluster A. In clusters D and F this is caused by a different adduct compared to cluster A, namely the dimers [2 M + H]^+^ for the DE *N*-oxides in cluster D and the sodium adduct [M + Na]^+^ in cluster F. The separation of the ME *N*-oxides in cluster C could be caused by differences in the MS2 spectra due to the presence of retronecine instead of heliotridine as necine base. The separation of the structures in cluster E compared to other retronecine based MC12s, can be explained by differences in the MS2 spectra, as they feature an epoxide in their necic acid. While the cosine score of 0.7 results in a high number of single nodes, lowering the cosine score leads to an even larger cluster A and to overly large number of connections inside the network. The original network can be accessed on GNPS (https://gnps.ucsd.edu/ProteoSAFe/status.jsp?task=cef65b240834462887c774c65b3fc6af), the modified MN, including names of the structures, is provided as S4.

#### Dereplication of the PASL against the GNPS libraries

In the second validation step, the compounds of the PASL were dereplicated against all current GNPS libraries (January 2025). First, the.mgf files of the spectra in the PASL were downloaded and used as input for the GNPS library search (https://gnps.ucsd.edu/ProteoSAFe/index.jsp?params=%7B%22workflow%22:%22MOLECULAR-LIBRARYSEARCH-V2%22,%22library_on_server%22:%22d.speclibs;%22%7D). The MS cluster option was disabled, the precursor mass tolerance was set to 0.025 Da, and the fragment ion mass tolerance to 0.02 Da. The threshold for the annotation was a minimum modified cosine score of 0.6, with minimum 6 peaks matched. In total 54 PAs had a match with spectra from the GNPS libraries (29 different compounds) (https://gnps.ucsd.edu/ProteoSAFe/status.jsp?task=c90414aaf1524fdf9058350104604183). However, only 26 of the annotations were correct, the other 29 PAs were incorrectly annotated as their (stereo)isomers (Table 1). This means, that the PASL increases the number of PAs in the GNPS libraries by 350%.

**Table 1:** PASL matched against existing GNPS libraries.

Compound (PASL)	Availability GNPS	Annotation GNPS	Quality GNPS	Score^a^	Difference	Quality PASL^b^
18-Hydroxyspartioidine	No	Erucifoline	Bronze	0.90	isomer	**Gold**
7-O-Acetylintermedine	No	7-Acetyllycopsamine	Bronze	0.97	epimers	**Gold**
7-O-Acetyllycopsamine	Yes	7-Acetyllycopsamine	Bronze	0.97		**Gold**
Echimidine	Yes	Echimidine	Bronze	0.97		**Gold**
Echimidine *N*-oxide	Yes	Echimidine *N*-oxide	Bronze	0.99		**Gold**
Echinatine	No	Rinderine	Bronze	0.89	epimers	**Gold**
Echinatine *N*-oxide	No	Intermedine *N*-oxide	Bronze	0.95	diastereomers	**Gold**
Epi-Jacobine	No	Jacobine	Bronze	0.95	epimers	**Gold**
Epi-Jacobine *N*-oxide	No	Jacobine *N*-oxide	Bronze	0.91	epimers	**Gold**
Erucifoline	Yes	Erucifoline	Bronze	0.96		**Gold**
Erucifoline *N*-oxide	Yes	Erucifoline *N*-oxide	Bronze	0.99		**Gold**
Europine	Yes	Europine	Bronze	0.97		**Gold**
Heliosupine	No	Echimidine	Bronze	0.88	diastereomers	**Gold**
Heliosupine *N*-oxide	No	Echimidine *N*-oxide	Bronze	0.68	diastereomers	**Gold**
Heliotrine	Yes	Heliotrine	Bronze	0.98		**Gold**
Heliotrine *N*-oxide	Yes	Heliotrine *N*-oxide	Bronze	1.00		**Gold**
Indicine	No	Intermedine	Bronze	1.00	epimers	**Gold**
Indicine *N*-oxide	No	Lycopsamine *N*-oxide	Bronze	1.00	epimers	**Gold**
Integerrimine	No	Senecionine	Bronze	0.94	cis/trans	**Gold**
Integerrimine *N*-oxide	No	Senecivernine *N*-oxide	Bronze	0.99	isomers	**Gold**
Intermedine	Yes	Intermedine	Bronze	1.00		**Gold**
Intermedine *N*-oxide	Yes	Lycopsamine *N*-oxide	Bronze	1.00	epimers	**Gold**
Jacobine	Yes	Jacobine	Bronze	0.95		**Gold**
Jacobine *N*-oxide	Yes	Jacobine *N*-oxide	Bronze	0.99		**Gold**
Jacozine *N*-ox	No	Erucifoline *N*-oxide	Bronze	0.62	isomers	**Bronze**
Lasiocarpine	Yes	Lasiocarpine	Bronze	0.98		**Gold**
Lasiocarpine *N*-oxide	Yes	Lasiocarpine *N*-oxide	Bronze	0.98		**Gold**
Lycopsamine	Yes	Intermedine	Bronze	1.00	epimers	**Gold**
Lycopsamine *N*-oxide	Yes	Lycopsamine *N*-oxide	Bronze	1.00		**Gold**
Merepoxine	No	Retrorsine	Bronze	0.82	isomers	**Gold**
Merepoxine *N*-oxide	No	Jacobine *N*-oxide	Bronze	0.82	isomers	**Gold**
Monocrotaline	Yes	Monocrotaline	Bronze	0.98		**Gold**
Monocrotaline *N*-oxide	Yes	Monocrotaline *N*-oxide	Bronze	0.98		**Gold**
Neosenkirkine	No	Senkirkine	Bronze	0.94	cis/trans	**Bronze**
Platyphylline	Yes	Platyhylline	Gold	0.98		**Gold**
Retrorsine	Yes	Retrorsine	Bronze	0.97		**Gold**
Retrorsine *N*-oxide	Yes	Retrorsine *N*-oxide	Bronze	0.98		**Gold**
Riddelliine	No	Erucifoline	Bronze	0.88	isomers	**Gold**
Riddelliine *N*-oxide	No	Erucifoline *N*-oxide	Bronze	0.87	isomers	**Gold**
Rinderine	Yes	Intermedine	Bronze	0.90	epimers	**Gold**
Rinderine *N*-oxide	Yes	Intermedine *N*-oxide	Bronze	0.94	epimers	**Gold**
Senecionine	Yes	Senecionine	Bronze	0.96		**Gold**
Senecionine *N*-oxide	Yes	Senecionine *N*-oxide	Bronze	0.99		**Gold**
Seneciphylline	Yes	Seneciphylline	Bronze	0.97		**Gold**
Seneciphylline *N*-oxide	Yes	Seneciphylline *N*-oxide	Bronze	0.9		**Gold**
Senecivernine	Yes	Senecionine	Bronze	0.94	isomers	**Gold**
Senecivernine *N*-oxide	Yes	Senecivernine *N*-oxide	Bronze	1.00		**Gold**
Senkirkine	Yes	Senkirkine	Bronze	0.94		**Gold**
Spartioidine	No	Seneciphylline	Bronze	0.97	cis/trans	**Gold**
Spartioidine *N*-oxide	No	Seneciphylline *N*-oxide	Bronze	0.90	isomers	**Gold**
Trachelanthamine *N*-oxide	Synonym	Heliocurassavicine *N*-oxide	Gold	0.98	isomers	Gold
Trichodesmine	Yes	Trichodesmine	Bronze	0.96		**Gold**
Usaramine	Yes	Retrorsine	Bronze	0.95	cis/trans	**Gold**
Usaramine *N*-oxide	No	Retrorsine *N*-oxide	Bronze	0.88	diastereomers	**Gold**

In bold letters the spectra with improved quality with the PASL, in underline annotations as isomer even though standard is available.

^a^Modified cosine score, ^b^Quality of spectra as classified in GNPS. Gold spectra are derived from analytical standards, bronze spectra from crude plant extracts.

Due to limitations in the approach of dereplicating the PASL against existing libraries, such as missing compounds due to e.g. different collision energies or different MS instrumentation, an additional comparison was performed. The planar InChI-Keys of all PAs in the PASL were manually checked against the cleaned-up GNPS libraries (https://external.gnps2.org/gnpslibrary, accessed on 1 April 2025). A of total 32 PAs compounds were available as high resolution MS2 spectra in the GNPS libraries. It should be noted that only 19 of those are regulated in the EU. Therefore, 16 of the 35 in the EU regulated PAs were not yet available in these open-access libraries (Table 1). One PA spectrum from the libraries was excluded, as it was recorded using low resolution MS. For 17 InChIKeys not the right compound itself, but its isomer was assigned. The PA otosenine could not be found using its InChIKey or exact precursor mass, while it could be found by its name. Closer inspection showed this happened due to an error in the uploaded molecular formula and structure of the uploaded molecule. When reconstructing the structure of the molecule in ChemDraw based on the provided SMILES code, a missing CH_2_ group was revealed. This emphasizes the importance of a thorough validation of the uploaded spectral libraries and highlights the risk of incorrect entries in open-access libraries.

The incorrect annotation of compounds as their isomers is a general issue with the HRMS analysis of stereoisomers, which fragment in the same pattern and are therefore not distinguishable by their MS2 spectra^20^. While FBMN in GNPS can sometimes differentiate between isomers if they elute on different RTs, corresponding nodes will often be wrongly annotated as the same compound due to the similar MS2 spectra. The only way to resolve this issue without applying other techniques is to manually review the results and correct the FBMN based on the RTs. However, in some cases the structure similarity of the compounds is so high, that they cannot be distinguished based on RT. For those epimers the application of other orthogonal separation approaches, such as Ion Mobility MS, or complementary annotation techniques such as NMR would be beneficial.

We provided the RTs of the method used for the development of this database in S2. The chromatographic settings and gradient of the method correspond to those developed and routinely applied in our laboratory for determining PAs in food and feed^30^.

#### FBMN on Jacobaea gnaphalioides and Heliotropium europaeum sample

In the third validation step, sample extracts of early stem leaves from 12-week-old *Jacobeae gnaphalioides* and *Heliotropium europaeum* were dereplicated using the PASL as reference library in FBMN. These species were selected as their PA profiles are known to contain complementary classes of PAs. The samples were analyzed under the same conditions as the PA standards to allow the annotation at the highest possible identification level on the Schymanski score. More than 20 compounds were putatively identified across the two species by using the PASL, as reference for the FBMN.

**Fig. 4:**
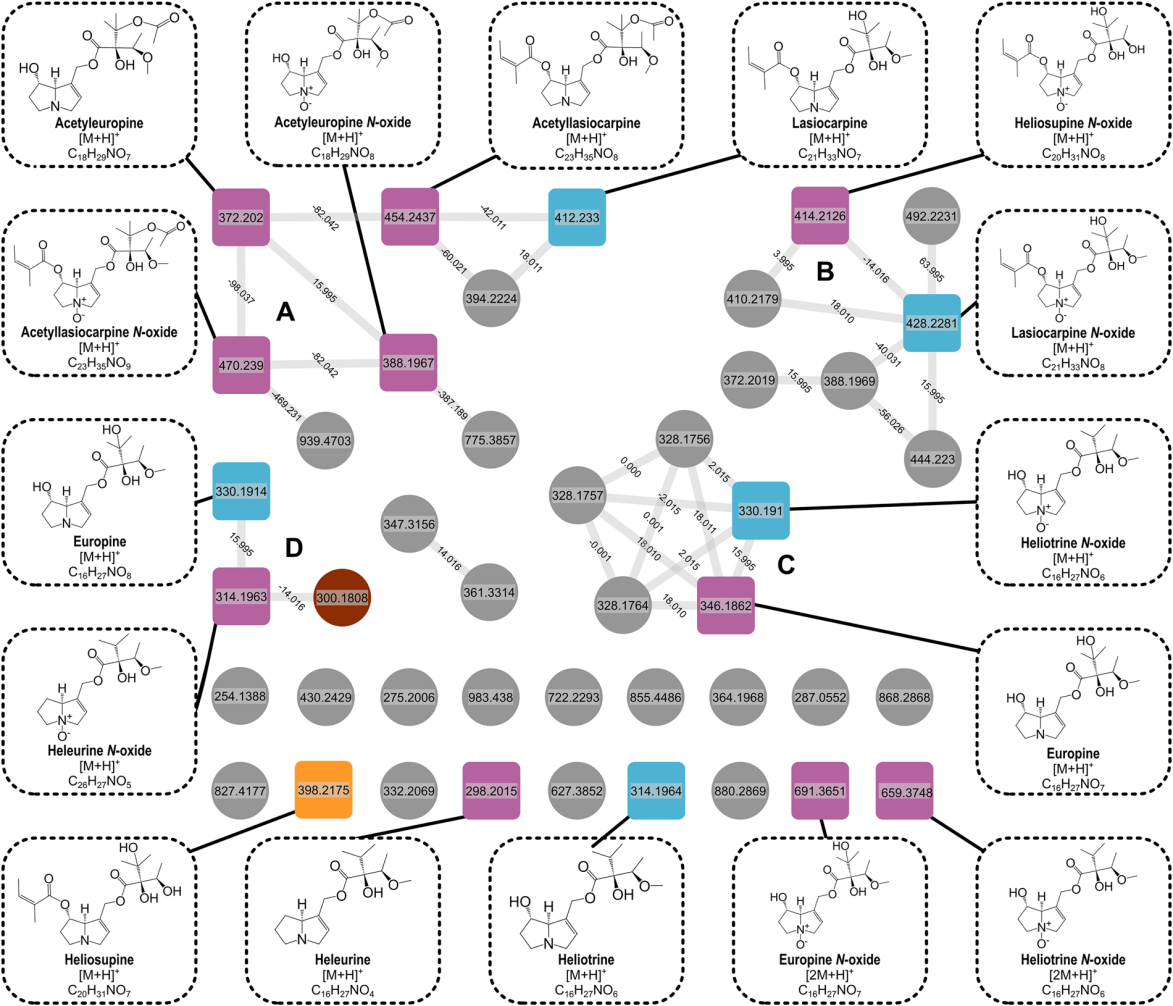
Feature-based molecular network of Heliotropium euopaeum, annotated with GNPS libraries and PASL. Squared nodes are PAs correctly annotated by the PASL, round nodes are not annotated as PA by the PASL. The color of the nodes indicates if GNPS libraries annotated PAs correctly (light blue), as isomers (orange), incorrectly (brown) or were absent in the GNPS libraries (purple). Network created in GNPS after data preprocessing in MZmine. Visualized with Cytoscape.

**Fig. 5:**
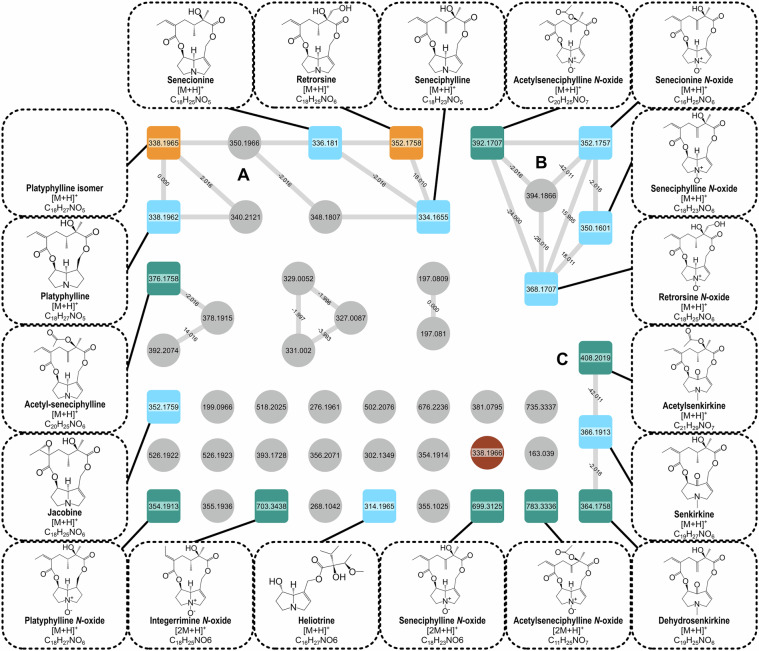
Feature-based molecular network of Jacobaea gnaphalioides, annotated with GNPS libraries and PASL. Squared nodes are PAs correctly annotated by the PASL, round nodes are not annotated as PA by the PASL. The color of the nodes indicates if GNPS libraries annotated PAs correctly (light blue), as isomers (orange), incorrectly (brown) or were absent in the GNPS libraries (teal). Network created in GNPS after data preprocessing in MZmine. Visualized with Cytoscape.

In the molecular networks, experimental data from *H. europaeum* are displayed as violet (Fig. 4) and *J. gnaphalioides* as teal (Fig. 5). In both networks the darker, squared nodes represent matches with the PASL. The nodes representing annotated PAs were partly clustered depending on the both the necine base and structure of the molecule, sorting the PAs into their different classes (Fig. 2). However, it is important to mention that there are also many single nodes not connected to their classes, which might be a result of using only one datafile as input and setting the cosine score high. Additional to the library matching, the confidence levels were assigned by following Schymanski *et al*.^28^ with most compounds achieving level 1 due to their exact match with an analytical standard used for the construction of the PASL. Their level was confirmed by RT and spectral matching (Table 2).

**Table 2:** PAs annotated in *H. europaeum* using the PASL.

Annotated PA	Precursor mass	*m/z* error [ppm]	Quality GNPS^c^	Reference
5′-acetyleuropine	372.2020	0.82	Bronze	^31,32,34^
5′-acetyleuropine *N*-oxide	388.1967	0.24	Bronze	^34^
5′-acetyllasiocarpine	454.2437	0.47	Bronze	^31,34^
5′-acetyllasiocarpine *N*-oxide	470.2390	1.10	Bronze	^32,34^
Europine	330.1914	0.92	Gold	^31–34^
Europine *N*-oxide	346.1862	0.53	Gold	^33,34^
Heleurine	298.2015	0.72	Bronze	^31,32^
Heleurine *N*-oxide	314.1963	0.29	Bronze	
Heliosupine	398.2175	0.54	Gold	^31,33,34^
Heliosupine *N*-oxide	414.2126	0.96	Gold	^33,34^
Heliotrine	314.1964	0.68	Gold	^31–34^
Heliotrine *N*-oxide	330.1910	0.28	Gold	^32–34^
Lasiocarpine	412.2330	0.00	Gold	^31–34^
Lasiocarpine *N*-oxide	428.2281	0.43	Gold	^33,34^
Echinatine/Rinderine^a,b^	300.1810	0.61	Gold	^31,33,34^
Echinatine *N*-oxide/Rinderine *N*-oxide^a,b^	316.1760	0.29	Gold	^33,34^
Intermedine *N*-oxide^a^	316.1760	0.29	Gold	
Trachelanthamine^a^	286.2010	0.32	Gold	
Trachelanthamine *N*-oxide^a^	302.1960	0.30	Gold	

^a^not visualized in network Fig. 5, ^b^Isomers with close retention time, ^c^Quality of spectra as classified in GNPS. Gold spectra are derived from analytical standards, bronze spectra from crude plant extracts.

In *H. europaeum*, the main cluster (cluster A) is predominantly composed of diesters with a retronecine or heliotridine base and modified with an acetyl group. The exception is lasiocarpine, which is connected to its corresponding acetyllasiocarpine. The smaller cluster (cluster B) represents *N*-oxide diesters. However, only two of the seven connected compounds could be annotated using the PASL, implicating that the sample contains more PA-like structures. This is similar to cluster C, which comprises ME *N*-oxides and three unknown, but closely related structures. Generally, all PAs annotated in *H. europaeum* are mono- or diesters, but no necine bases or macroesters are present. Some nodes, such as heliotrine *N*-oxide and europine *N*-oxide are identified twice, but with different precursors. This highlights the advantage of PASL to include for some compounds not only the [M + H]^+^, but also other adducts. Overall, eleven of the 16 annotated PAs could be annotated only with the PASL, as the libraries on GNPS do not contain the corresponding standards. The GNPS libraries annotated heliosupine as its cis/trans-isomer echimidine. An interesting case is node 300.1808 in cluster D. This was incorrectly annotated as rinderine by the GNPS libraries but not identified by the PASL. The PASL contains the spectra of rinderine, however, the cosine score was probably below the threshold. Additionally, the RT is not correct, which indicates that the compound is probably not rinderine. The small size of the clusters is caused by the strict settings in GNPS, which were necessary to achieve multiple clusters instead of one large network due to the high similarity in the structures.

In *J. gnaphalioides*, the cluster (A) is composed of MC12 with a retronecine or platynecine base. Additional to platyphylline, there is an isomer that was, based on the (RT), annotated incorrectly. The smaller cluster (B) consists mainly of the corresponding *N*-oxides to (A). However, platyphylline *N*-oxide is missing, and can be found as a single node. Cluster C represents the group of senkirkine type PAs. Six of the nodes in the network are presented as single nodes, indicating that their MS2 spectra are not closely related to that of other PAs. For acetylseneciphylline *N*-oxide, seneciphylline *N*-oxide and integerrimine *N*-oxide, this is caused by the different adduct [2 M + H]^+^, that primarily occurs for the *N*-oxide form of PAs. Jacobine differs from the other 12MC in *J. gnaphalioides* by an epoxide group in its necic base. Of the 19 annotated nodes, nine could be correctly annotated using GNPS libraries. Two nodes (*m/z* 352 and *m/z* 338) were annotated as the isomer by GNPS libraries, but correctly by the PASL. Also in this network there is one compound that was incorrectly identified as platyphylline, even though it is not recognized as such by the PASL and there is a 10 ppm mass error. By only using the PASL the compound cannot be determined as a PA.

The PA profile annotated in *H. europaeum* is overall the same as reported by previous studies (Table 2)^31–33^. A few exceptions could not be found in this network, such as supinine or echinatine. However, this is caused by either not having standards for the compounds (supinine) or by the strict conditions such for pre-processing such as a high minimum feature height (echinatine). When the restrictions are eased, more compounds could be annotated (S6, Table 2). In this paper, we decided to apply stricter criteria for the FBMN, to improve the visualization clarity and to prevent the figure from becoming overly large and cluttered. Compared to *H. europaeum*, the PA profile of *J. gnaphalioides* has not yet been studied in detail in the past. The only reported study was done on the same *J. gnaphalioides* sample using LC-MS/MS^29^. The annotations obtained using the PASL match those determined previously by Chen *et al*. (S7, S8). In the network of *J*. *gnaphalioides* the ME heliotrine was annotated, even though this PA is not produced by *Jacobaea* species. However, it was used as an internal standard when preparing the extracts and can therefore be detected in this specific sample.

Overall, the results of the three-step validation process demonstrate the importance of our PASL for the annotation of known and new PAs in samples. As the first spectral library dedicated to only PAs, the PASL includes 79 commercially available standards, 5 semi-synthesized compounds, and 18 dereplicated PAs, making it ideal for metabolomics studies. Compared to the publicly available spectral libraries, which do not contain many PAs that are important for food safety, the PASL provides a great selection of PA MS2 reference spectra. Due to the high number of stereoisomers, the MS2 spectra alone are not always enough to correctly annotate each PA, as they show identical fragmentation. The only way to resolve this issue without using orthogonal techniques is to take the RT into account. In the metadata we provide the RTs and structures of all PAs included in this library, to facilitate a better annotation. However, in order to baseline separate all isomers, a very long LC method would be necessary. Therefore, the annotation especially of closely eluting, stereoisomers should always be tentatively. We demonstrated its utility in the annotation of PA in two plant extracts, resulting in a higher number of annotations and better accuracy. Furthermore, we aim to enlarge the library by the synthesis of a series of selected PAs and the detailed analysis thereof. The availability of spectral libraries such as the PASL is crucial in the field of metabolomics, as they enable both computational approaches such as FBMN and basic spectral library matching without requiring access to reference standards.

## Supplementary information

Supplementary material PASL data descriptor: Development of a High-Resolution Tandem Mass Spectral Library for Pyrrolizidine Alkaloids (PASL)

## Data Availability

The spectral library is available on GNPS under the accession numbers CCMSLIB00014205782 to CCMSLIB00014205946 (https://gnps.ucsd.edu/ProteoSAFe/gnpslibrary.jsp?library=PYRROLIZIDINE-ALKALOID-SPECTRAL-LIBRARY). The dataset has been deposited to MassIVE under the accession number MSV000098010^27^.

## Code availability

No custom code was used. The workflow used for the creation of the spectral library is available on GNPS.
