# Supplementary material for: Development of a High-Resolution Tandem Mass Spectral Library for Pyrrolizidine Alkaloids (PASL)
Source: Sci Data. 2025 Oct 20;12:1654. doi: 10.1038/s41597-025-05940-7 (PMC12537953; doi:10.1038/s41597-025-05940-7)
Supplement: Supplementary file 1 — Supplementary material PASL data descriptor: Development of a High-Resolution Tandem Mass Spectral Library for Pyrrolizidine Alkaloids (PASL) [file 41597_2025_5940_MOESM1_ESM.docx]

# Supplementary material PASL data descriptor

Development of a High-Resolution Tandem Mass Spectral Library for Pyrrolizidine Alkaloids (PASL)

## Authors

Leonie V. Straub^1,2^, Patrick P.J. Mulder^1,†^, Han Zuilhof^2,3^, Federico Padilla Gonzalez^1^, Laura Righetti*^,1,2^

## Affiliations

^1^ Wageningen Food Safety Research, Wageningen University & Research, Wageningen, The Netherlands

^2^ Laboratory of Organic Chemistry, Wageningen University & Research, Wageningen, The Netherlands

^3^ College of Biological and Chemical Engineering, Jiaxing University, Jiaxing 314001, China

^†^Deceased

Corresponding authors: Dr. Laura Righetti (laura.righetti@wur.nl) and Dr. Federico Padilla Gonzales (federico.padillagonzalez@wur.nl)

## S1: incorrect structure based on SMILES

Compounds drawn based on the JECFA nomenclature in ChemSketch. Isomeric SMILES created in ChemSketch based on structure. Structures were re-created based on the SMILES in ChemDraw. The stereochemistry and orientation were kept and no changes were made. The relevant part of the molecule is highlighted, in pink, the structural deviation of 7-Acetyl-lycopsamine N-oxide.


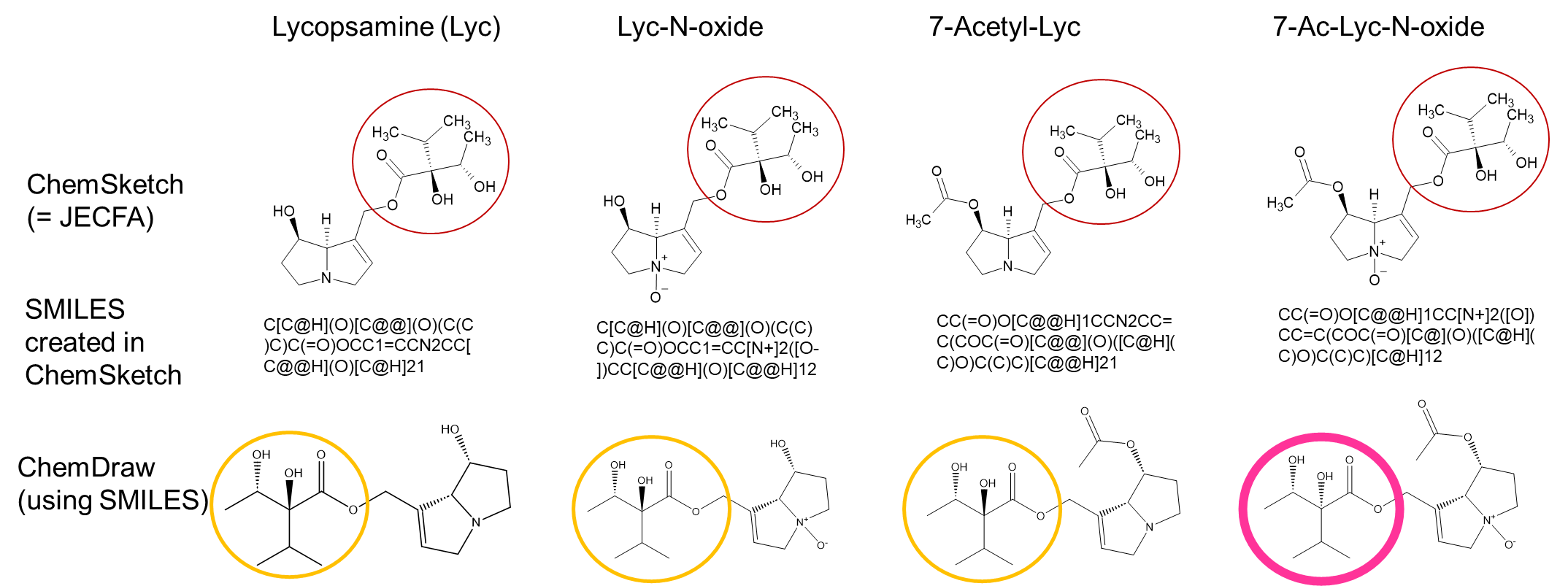


## S2: Pyrrolizidine Alkaloids in PASL

Abbreviations: retention time (RT), retronecine (R), heliotridine (H), platyphylline (P), otonecine (O), trachelanthamidine (T), rosmarinecine (D), monoester (ME), diester (DE), macrocyclic esters with 12-membered ring (MC12), macrocyclic esters with 11 membered ring (MC11) and necine bases (NB)

| **Compound** | **Adduct** | ***m/z*** | **RT** | **Base** | **Type** | **Isomeric SMILES** | **InChI** | **CAS-number** | **Origin** | **Genus/species** | **Quality** | **Structure** |
| --- | --- | --- | --- | --- | --- | --- | --- | --- | --- | --- | --- | --- |
| **18-Hydroxyspartioidine** | [[M+H]+]+ [M+Na]+ | 350.1598 372.1417 | 8.1 | R | 12MC | O[C@@]1(CO)C(=O)OCC2=CCN3CC[C@H](OC(=O)/C(CC1=C)=C/C)[C@@H]32 | InChI=1S/C18H23NO6/c1-3-12-8-11(2)18(23,10-20)17(22)24-9-13-4-6-19-7-5-14(15(13)19)25-16(12)21/h3-4,14-15,20,23H,2,5-10H2,1H3/b12-3+/t14-,15-,18+/m0/s1 | N/A | Commercial | N/A | 1 | 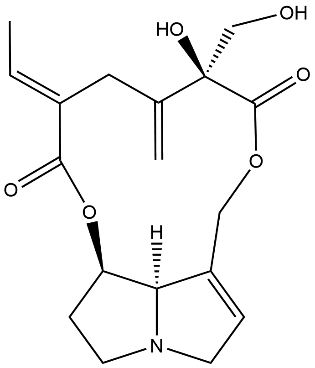 |
| **7-O-acetylintermedine** | [M+H]+ [M+Na]+ | 342.1911 364.1731 | 9.43 | R | DE | CC(=O)O[C@@H]1CCN2CC=C(COC(=O)[C@@](O)([C@@H](C)O)C(C)C)[C@@H]21 | InChI=1S/C17H27NO6/c1-10(2)17(22,11(3)19)16(21)23-9-13-5-7-18-8-6-14(15(13)18)24-12(4)20/h5,10-11,14-15,19,22H,6-9H2,1-4H3/t11-,14-,15-,17?/m1/s1 | 74243-01-09 | Commercial | N/A | 1 | 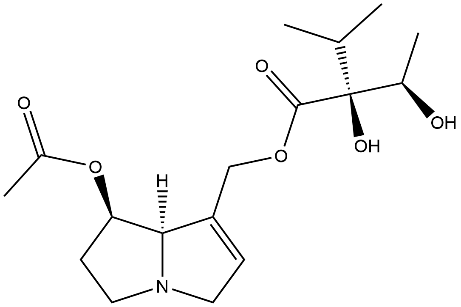 |
| **7-O-acetylintermedine N-oxide** | [M+H]+ [2M+H]+ | 358.1860 715.3649 | 6.41 | R | DE | CC(=O)O[C@@H]1CC[N+]2(O)CC=C(COC(=O)[C@](O)([C@H](C)O)C(C)C)[C@H]12 | InChI=1S/C17H28NO7/c1-10(2)17(22,11(3)19)16(21)24-9-13-5-7-18(23)8-6-14(15(13)18)25-12(4)20/h5,10-11,14-15,19,22-23H,6-9H2,1-4H3/q+1/t11-,14+,15+,17?,18?/m0/s1 | 685132-59-6 | Commercial | N/A | 1 | 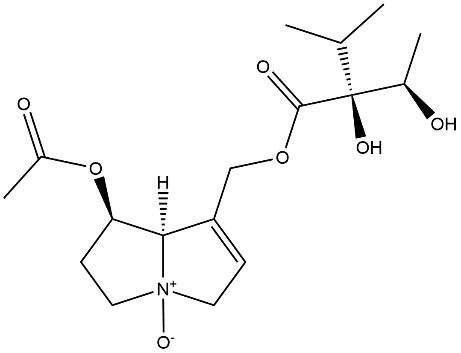 |
| **7-O-acetyllycopsamine** | [M+H]+ [M+Na]+ | 342.1911 364.1731 | 9.49 | R | DE | CC(=O)O[C@@H]1CCN2CC=C(COC(=O)[C@@](O)([C@H](C)O)C(C)C)[C@@H]21 | InChI=1S/C17H27NO6/c1-10(2)17(22,11(3)19)16(21)23-9-13-5-7-18-8-6-14(15(13)18)24-12(4)20/h5,10-11,14-15,19,22H,6-9H2,1-4H3/t11-,14+,15+,17?/m0/s1 | 73544-48-6 | Commercial | N/A | 1 | 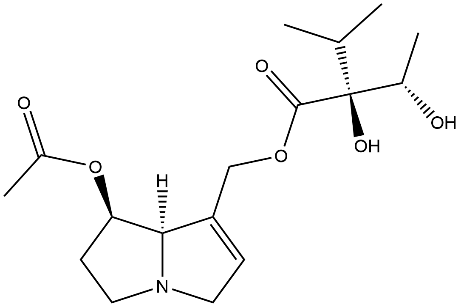 |
| **7-O-acetyllycopsamine N-oxide** | [M+H]+ | 358.1860 | 6.38 | R | DE | CC(=O)O[C@@H]1CC[N+]2(O)CC=C(COC(=O)[C@](O)([C@@H](C)O)C(C)C)[C@H]12 | InChI=1S/C17H28NO7/c1-10(2)17(22,11(3)19)16(21)24-9-13-5-7-18(23)8-6-14(15(13)18)25-12(4)20/h5,10-11,14-15,19,22-23H,6-9H2,1-4H3/q+1/t11-,14-,15-,17?,18?/m1/s1 | 685132-58-5 | Commercial | N/A | 1 | 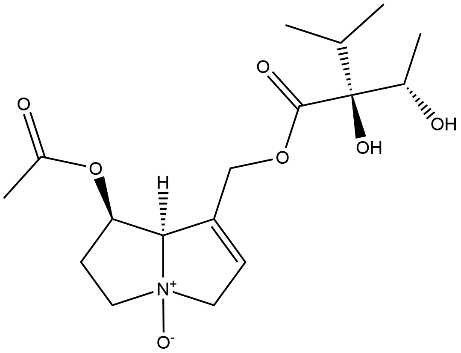 |
| **Echimidine** | [M+H]+ [M+Na]+ | 398.2173 420.1993 | 11.01 | R | DE | CC(C)(O)C(O)(C(=O)OCC1=CCN2CC[C@@H](OC(=O)\C(C)=C/C)[C@H]21)[C@H](C)O | InChI=1S/C20H31NO7/c1-6-12(2)17(23)28-15-8-10-21-9-7-14(16(15)21)11-27-18(24)20(26,13(3)22)19(4,5)25/h6-7,13,15-16,22,25-26H,8-11H2,1-5H3/b12-6-/t13-,15+,16+,20?/m0/s1 | 520-68-3 | Commercial | N/A | 1 | 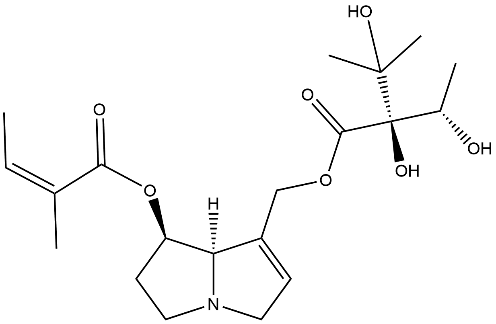 |
| **Echimidine N-oxide** | [M+H]+ [2M+H]+ | 414.2122 827.4172 | 7.96 | R | DE | CC(C)(O)C(O)(C(=O)OCC1=CC[N+]2([O-])CC[C@@H](OC(=O)\C(C)=C/C)[C@@H]12)[C@H](C)O | InChI=1S/C20H31NO8/c1-6-12(2)17(23)29-15-8-10-21(27)9-7-14(16(15)21)11-28-18(24)20(26,13(3)22)19(4,5)25/h6-7,13,15-16,22,25-26H,8-11H2,1-5H3/b12-6-/t13-,15+,16+,20?,21?/m0/s1 | 41093-89-4 | Commercial | N/A | 1 | 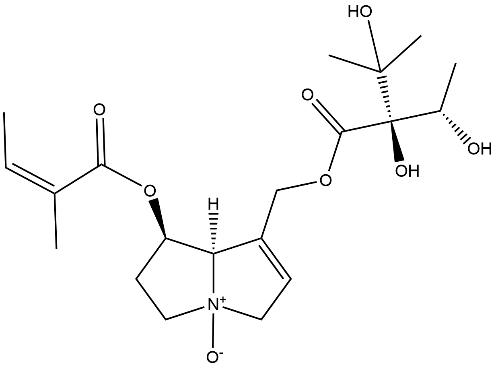 |
| **Echinatine** | [M+H]+ | 300.1805 | 6.86 | H | ME | C[C@H](O)[C@@](O)(C(C)C)C(=O)OCC1=CCN2CC[C@H](O)[C@H]21 | InChI=1S/C15H25NO5/c1-9(2)15(20,10(3)17)14(19)21-8-11-4-6-16-7-5-12(18)13(11)16/h4,9-10,12-13,17-18,20H,5-8H2,1-3H3/t10-,12-,13+,15?/m0/s1 | 480-83-1 | Commercial | N/A | 1 | 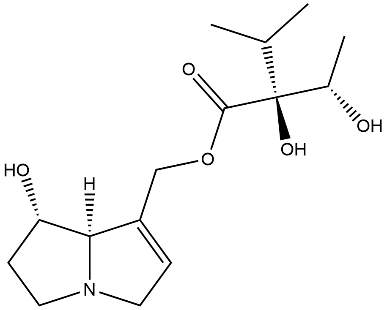 |
| **Echinatine N-oxide** | [M+H]+ [2M+H]+ | 316.1755 631.3437 | 4.21 | H | ME | C[C@H](O)[C@@](O)(C(C)C)C(=O)OCC1=CC[N+]2([O-])CC[C@H](O)[C@@H]12 | InChI=1S/C15H25NO6/c1-9(2)15(20,10(3)17)14(19)22-8-11-4-6-16(21)7-5-12(18)13(11)16/h4,9-10,12-13,17-18,20H,5-8H2,1-3H3/t10-,12-,13+,15?,16?/m0/s1 | 20267-93-0 | Commercial | N/A | 1 | 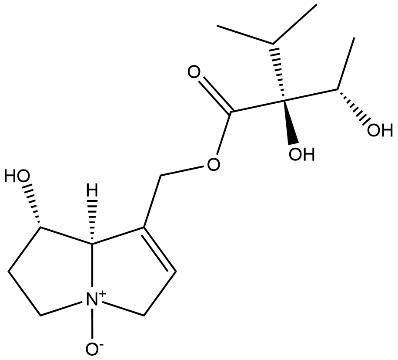 |
| **Echiumine** | [M+H]+ [M+Na]+ [M+K]+ | 382.2224 404.2044 420.1782 | 11.81 | R | DE | C[C@H](O)C(O)(C(=O)OCC1=CCN2CC[C@@H](OC(=O)\C(C)=C/C)[C@H]21)C(C)C | InChI=1S/C20H31NO6/c1-6-13(4)18(23)27-16-8-10-21-9-7-15(17(16)21)11-26-19(24)20(25,12(2)3)14(5)22/h6-7,12,14,16-17,22,25H,8-11H2,1-5H3/b13-6-/t14-,16+,17+,20?/m0/s1 | 633-16-9 | Commercial | N/A | 1 | 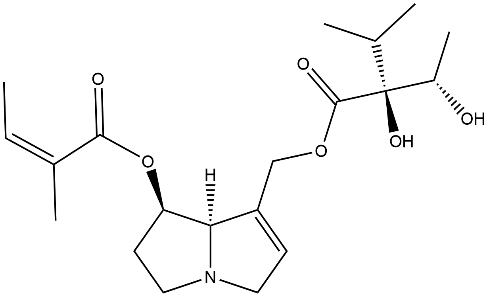 |
| **Echiumine N-oxide** | [M+H]+ [2M+H]+ | 398.2173 795.4274 | 9.78 | R | DE | C[C@H](O)C(O)(C(=O)OCC1=CC[N+]2([O-])CC[C@@H](OC(=O)\C(C)=C/C)[C@@H]12)C(C)C | InChI=1S/C20H31NO7/c1-6-13(4)18(23)28-16-8-10-21(26)9-7-15(17(16)21)11-27-19(24)20(25,12(2)3)14(5)22/h6-7,12,14,16-17,22,25H,8-11H2,1-5H3/b13-6-/t14-,16+,17+,20?,21?/m0/s1 | 685554-68-1 | Commercial | N/A | 1 | 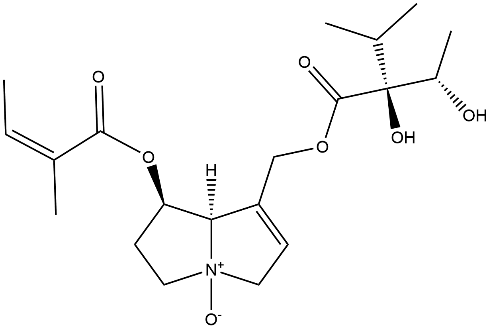 |
| **Epi-Jacobine** | [M+H]+ [M+Na]+ | 352.1755 374.1574 | 9.42 | R | 12MC | C[C@]1(O)C(=O)OCC2=CCN3CC[C@@H](OC(=O)[C@]4(C[C@H]1C)O[C@@H]4C)[C@H]32 | InChI=1S/C18H25NO6/c1-10-8-18(11(2)25-18)16(21)24-13-5-7-19-6-4-12(14(13)19)9-23-15(20)17(10,3)22/h4,10-11,13-14,22H,5-9H2,1-3H3/t10-,11-,13-,14-,17-,18-/m1/s1 | 6870-67-3 | Commercial | N/A | 1 | 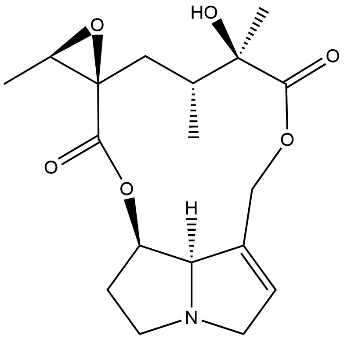 |
| **Epi-Jacobine N-oxide** | [M+H]+ [2M+H]+ | 368.1704 735.3335 | 6.22 | R | 12MC | C[C@]1(O)C(=O)OCC2=CC[N+]3([O-])CC[C@@H](OC(=O)[C@]4(C[C@H]1C)O[C@@H]4C)[C@@H]23 | InChI=1S/C18H25NO7/c1-10-8-18(11(2)26-18)16(21)25-13-5-7-19(23)6-4-12(14(13)19)9-24-15(20)17(10,3)22/h4,10-11,13-14,22H,5-9H2,1-3H3/t10-,11-,13-,14-,17-,18-,19?/m1/s1 | 38710-25-7 | Isolated | N/A | 1 | 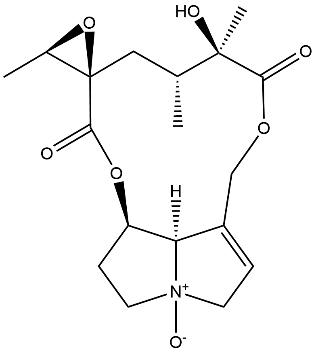 |
| **Erucifoline** | [M+H]+ | 350.1598 | 7.83 | R | 12MC | O=C1OCC2=CCN3CC[C@@H](OC(=O)\C(C[C@@]4(O[C@@]14C)CO)=C/C)[C@H]32 | InChI=1S/C18H23NO6/c1-3-11-8-18(10-20)17(2,25-18)16(22)23-9-12-4-6-19-7-5-13(14(12)19)24-15(11)21/h3-4,13-14,20H,5-10H2,1-2H3/b11-3-/t13-,14-,17+,18-/m1/s1 | 40158-95-0 | Commercial | N/A | 1 | 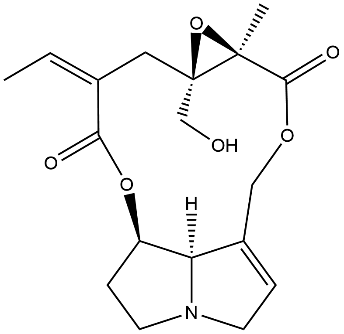 |
| **Erucifoline N-oxide** | [M+H]+ [2M+H]+ | 366.1547 731.3022 | 4.93 | R | 12MC | [O-][N+]12CC[C@H]3OC(=O)\C(C[C@@]4(O[C@@]4(C)C(=O)OCC(=CC1)[C@H]32)CO)=C/C | InChI=1S/C18H23NO7/c1-3-11-8-18(10-20)17(2,26-18)16(22)24-9-12-4-6-19(23)7-5-13(14(12)19)25-15(11)21/h3-4,13-14,20H,5-10H2,1-2H3/b11-3-/t13-,14-,17+,18-,19?/m1/s1 | 123864-94-8 | Commercial | N/A | 1 | 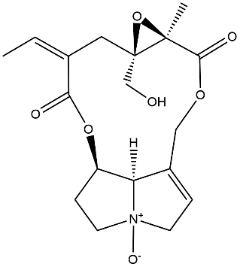 |
| **Europine** | [M+H]+ [M+Na]+ | 330.1911 352.1731 | 6.59 | H | ME | CO[C@H](C)[C@@](O)(C(=O)OCC1=CCN2CC[C@H](O)[C@H]21)C(C)(O)C | InChI=1S/C16H27NO6/c1-10(22-4)16(21,15(2,3)20)14(19)23-9-11-5-7-17-8-6-12(18)13(11)17/h5,10,12-13,18,20-21H,6-9H2,1-4H3/t10-,12+,13-,16?/m1/s1 | 570-19-4 | Commercial | N/A | 1 | 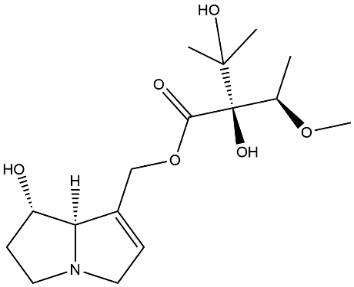 |
| **Europine N-oxide** | [M+H]+ [2M+H]+ | 346.1860 691.3648 | 4.02 | H | ME | CO[C@H](C)[C@@](O)(C(=O)OCC1=CC[N+]2([O-])CC[C@H](O)[C@@H]12)C(C)(O)C | InChI=1S/C16H27NO7/c1-10(23-4)16(21,15(2,3)20)14(19)24-9-11-5-7-17(22)8-6-12(18)13(11)17/h5,10,12-13,18,20-21H,6-9H2,1-4H3/t10-,12+,13-,16?,17?/m1/s1 | 65582-53-8 | Commercial | N/A | 1 | 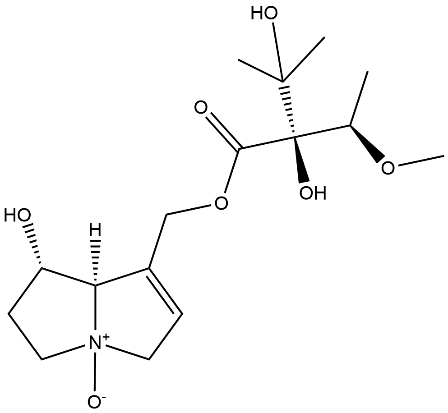 |
| **Heliosupine** | [M+H]+ [M+Na]+ | 398.2173 420.1993 | 10.9 | H | DE | CC(C)(O)[C@](O)(C(=O)OCC1=CCN2CC[C@H](OC(=O)\C(C)=C/C)[C@H]21)[C@@H](C)O | InChI=1S/C20H31NO7/c1-6-12(2)17(23)28-15-8-10-21-9-7-14(16(15)21)11-27-18(24)20(26,13(3)22)19(4,5)25/h6-7,13,15-16,22,25-26H,8-11H2,1-5H3/b12-6-/t13-,15+,16-,20?/m1/s1 | 32728-78-2 | Commercial | N/A | 1 | 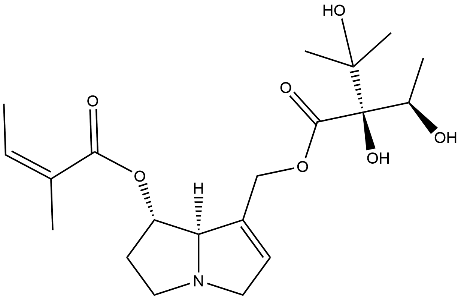 |
| **Heliosupine N-oxide** | [M+H]+ [2M+H]+ | 414.2122 827.4172 | 7.65 | H | DE | CC(C)(O)C(O)(C(=O)OCC1=CC[N+]2([O-])CC[C@H](OC(=O)\C(C)=C/C)[C@@H]12)[C@@H](C)O | InChI=1S/C20H31NO8/c1-6-12(2)17(23)29-15-8-10-21(27)9-7-14(16(15)21)11-28-18(24)20(26,13(3)22)19(4,5)25/h6-7,13,15-16,22,25-26H,8-11H2,1-5H3/b12-6-/t13-,15+,16-,20?,21?/m1/s1 | 31701-88-9 | Commercial | N/A | 1 | 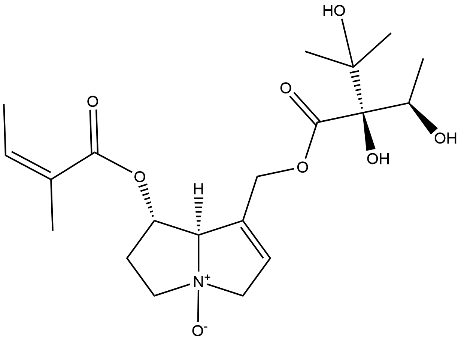 |
| **Heliotridine** | [M+H]+ | 156.1019 | 1.92 | H | FB | O[C@H]1CCN2CC=C(CO)[C@@H]21 | InChI=1S/C8H13NO2/c10-5-6-1-3-9-4-2-7(11)8(6)9/h1,7-8,10-11H,2-5H2/t7-,8+/m0/s1 | 520-63-8 | Commercial | N/A | 1 | 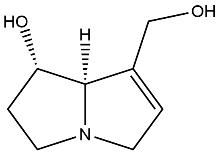 |
| **Heliotrine** | [M+H]+ | 314.1962 | 8.6 | H | ME | CO[C@@H](C)[C@@](O)(C(=O)OCC1=CCN2CC[C@H](O)[C@H]21)C(C)C | InChI=1S/C16H27NO5/c1-10(2)16(20,11(3)21-4)15(19)22-9-12-5-7-17-8-6-13(18)14(12)17/h5,10-11,13-14,18,20H,6-9H2,1-4H3/t11-,13-,14+,16+/m0/s1 | 303-33-3 | Commercial | N/A | 1 | 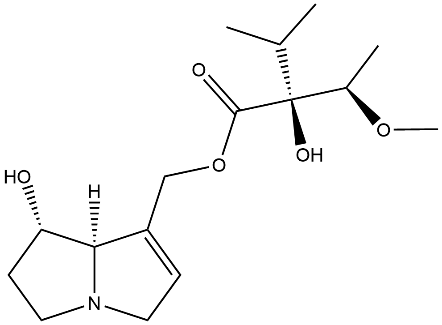 |
| **Heliotrine N-oxide** | [M+H]+ [2M+H]+ | 330.1911 659.3750 | 6 | H | ME | CO[C@H](C)[C@@](O)(C(=O)OCC1=CC[N+]2([O-])CC[C@H](O)[C@@H]12)C(C)C | InChI=1S/C16H27NO6/c1-10(2)16(20,11(3)22-4)15(19)23-9-12-5-7-17(21)8-6-13(18)14(12)17/h5,10-11,13-14,18,20H,6-9H2,1-4H3/t11-,13+,14-,16?,17?/m1/s1 | 6209-65-0 | Commercial | N/A | 1 | 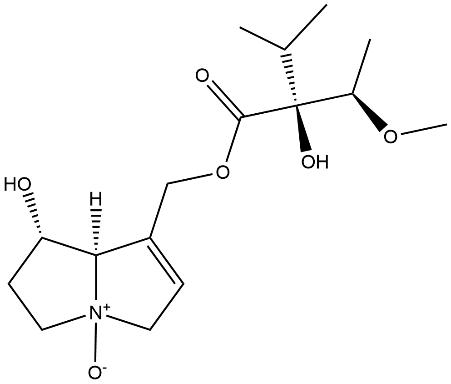 |
| **Indicine** | [M+H]+ | 300.1805 | 5.94 | R | ME | C[C@H](O)[C@@](O)(C(C)C)C(=O)OCC1=CCN2CC[C@@H](O)[C@H]21 | InChI=1S/C15H25NO5/c1-9(2)15(20,10(3)17)14(19)21-8-11-4-6-16-7-5-12(18)13(11)16/h4,9-10,12-13,17-18,20H,5-8H2,1-3H3/t10-,12+,13+,15?/m0/s1 | 480-82-0 | Commercial | N/A | 1 | 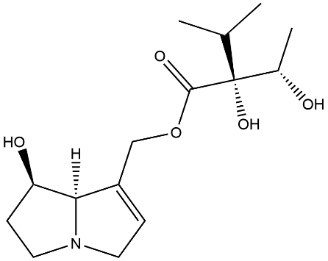 |
| **Indicine N-oxide** | [M+H]+ [2M+H]+ | 316.1755 631.3437 | 3.98 | R | ME | C[C@H](O)[C@](O)(C(C)C)C(=O)OCC1=CC[N+]2([O-])CC[C@@H](O)[C@@H]12 | InChI=1S/C15H25NO6/c1-9(2)15(20,10(3)17)14(19)22-8-11-4-6-16(21)7-5-12(18)13(11)16/h4,9-10,12-13,17-18,20H,5-8H2,1-3H3/t10-,12+,13+,15?,16?/m0/s1 | 41708-76-3 | Commercial | N/A | 1 | 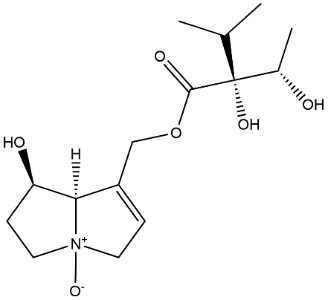 |
| **Integerrimine** | [M+H]+ | 336.1805 | 10.61 | R | 12MC | C[C@]1(O)C(=O)OCC2=CCN3CC[C@@H](OC(=O)/C(C[C@H]1C)=C/C)[C@H]32 | InChI=1S/C18H25NO5/c1-4-12-9-11(2)18(3,22)17(21)23-10-13-5-7-19-8-6-14(15(13)19)24-16(12)20/h4-5,11,14-15,22H,6-10H2,1-3H3/b12-4+/t11-,14-,15-,18-/m1/s1 | 480-79-5 | Commercial | N/A | 1 | 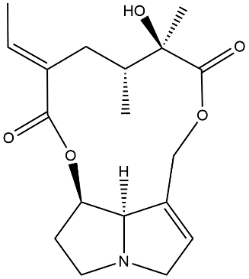 |
| **Integerrimine N-oxide** | [M+H]+ [2M+H]+ | 352.1755 703.3437 | 7 | R | 12MC | C[C@]1(O)C(=O)OCC2=CC[N+]3([O-])CC[C@@H](OC(=O)/C(C[C@H]1C)=C/C)[C@@H]23 | InChI=1S/C18H25NO6/c1-4-12-9-11(2)18(3,22)17(21)24-10-13-5-7-19(23)8-6-14(15(13)19)25-16(12)20/h4-5,11,14-15,22H,6-10H2,1-3H3/b12-4+/t11-,14-,15-,18-,19?/m1/s1 | 85955-28-8 | Commercial | N/A | 1 | 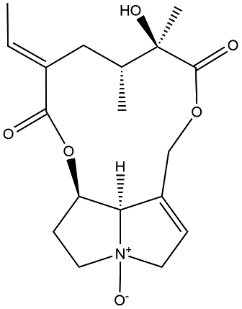 |
| **Intermedine** | [M+H]+ | 300.1805 | 5.85 | R | ME | C[C@@H](O)[C@@](O)(C(C)C)C(=O)OCC1=CCN2CC[C@@H](O)[C@H]21 | InChI=1S/C15H25NO5/c1-9(2)15(20,10(3)17)14(19)21-8-11-4-6-16-7-5-12(18)13(11)16/h4,9-10,12-13,17-18,20H,5-8H2,1-3H3/t10-,12-,13-,15?/m1/s1 | 10285-06-0 | Commercial | N/A | 1 | 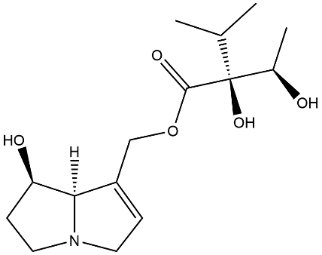 |
| **Intermedine N-oxide** | [M+H]+ [2M+H]+ | 316.1755 631.3437 | 3.8 | R | ME | C[C@@H](O)[C@@](O)(C(C)C)C(=O)OCC1=CC[N+]2([O-])CC[C@@H](O)[C@@H]12 | InChI=1S/C15H25NO6/c1-9(2)15(20,10(3)17)14(19)22-8-11-4-6-16(21)7-5-12(18)13(11)16/h4,9-10,12-13,17-18,20H,5-8H2,1-3H3/t10-,12-,13-,15?,16?/m1/s1 | 95462-14-9 | Commercial | N/A | 1 | 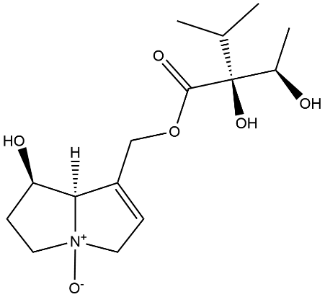 |
| **Jacobine** | [M+H]+ [M+Na]+ | 352.1755 374.1574 | 8.26 | R | 12MC | C[C@]1(O)C(=O)OCC2=CCN3CC[C@@H](OC(=O)[C@@]4(C[C@H]1C)O[C@H]4C)[C@H]32 | InChI=1S/C18H25NO6/c1-10-8-18(11(2)25-18)16(21)24-13-5-7-19-6-4-12(14(13)19)9-23-15(20)17(10,3)22/h4,10-11,13-14,22H,5-9H2,1-3H3/t10-,11+,13-,14-,17-,18+/m1/s1 | 6870-67-3 | Commercial | N/A | 1 | 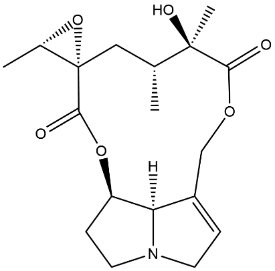 |
| **Jacobine N-oxide** | [M+H]+ [2M+H]+ | 368.1704 735.3335 | 4.84 | R | 12MC | C[C@]1(O)C(=O)OCC2=CC[N+]3([O-])CC[C@@H](OC(=O)[C@@]4(C[C@H]1C)O[C@H]4C)[C@@H]23 | InChI=1S/C18H25NO7/c1-10-8-18(11(2)26-18)16(21)25-13-5-7-19(23)6-4-12(14(13)19)9-24-15(20)17(10,3)22/h4,10-11,13-14,22H,5-9H2,1-3H3/t10-,11+,13-,14-,17-,18+,19?/m1/s1 | 38710-25-7 | Commercial | N/A | 1 | 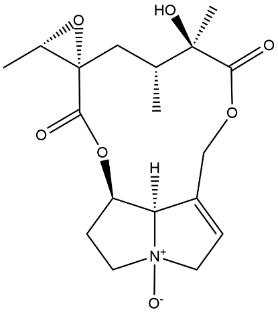 |
| **Jacoline** | [M+H]+ | 370.1860 | 5.87 | R | 12MC | C[C@]1(O)C(=O)OCC2=CCN3CC[C@@H](OC(=O)[C@](O)(C[C@H]1C)[C@H](O)C)[C@H]32 | InChI=1S/C18H27NO7/c1-10-8-18(24,11(2)20)16(22)26-13-5-7-19-6-4-12(14(13)19)9-25-15(21)17(10,3)23/h4,10-11,13-14,20,23-24H,5-9H2,1-3H3/t10-,11-,13-,14-,17-,18+/m1/s1 | 480-76-2 | Commercial | N/A | 1 | 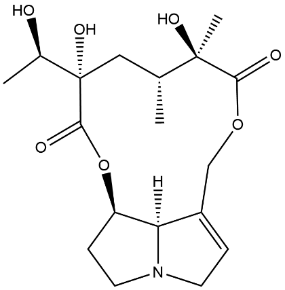 |
| **Jacoline N-oxide** | [M+H]+ [2M+H]+ | 386.1810 771.3546 | 2.92 | R | 12MC | C[C@]1(O)C(=O)OCC2=CC[N+]3([O-])CC[C@H](OC(=O)[C@](O)(C[C@H]1C)C(O)C)[C@H]23 | InChI=1S/C18H27NO8/c1-10-8-18(24,11(2)20)16(22)27-13-5-7-19(25)6-4-12(14(13)19)9-26-15(21)17(10,3)23/h4,10-11,13-14,20,23-24H,5-9H2,1-3H3/t10-,11?,13+,14+,17-,18+,19?/m1/s1 | 1148039-73-9 | Commercial | N/A | 1 |  |
| **Jaconine** | [M+H]+ | 388.1521 | 9.43 | R | 12MC | C[C@]1(O)C(=O)OCC2=CCN3CC[C@H](OC(=O)[C@](O)(C[C@H]1C)C(C)Cl)[C@@H]32 | InChI=1S/C18H26ClNO6/c1-10-8-18(24,11(2)19)16(22)26-13-5-7-20-6-4-12(14(13)20)9-25-15(21)17(10,3)23/h4,10-11,13-14,23-24H,5-9H2,1-3H3/t10-,11?,13+,14+,17-,18+/m1/s1 | 480-75-1 | Commercial | N/A | 1 | 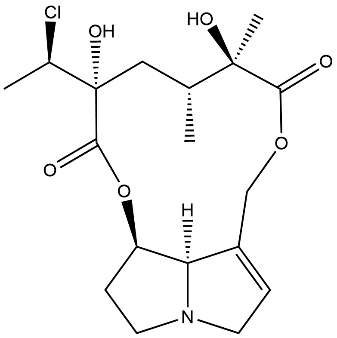 |
| **Jaconine N-oxide** | [M+H]+ [2M+H]+ | 404.1471 807.2868 | 5.43 | R | 12MC | C[C@]1(O)C(=O)OCC2=CC[N+]3([O-])CC[C@@H](OC(=O)[C@](O)(C[C@H]1C)C(C)Cl)[C@@H]23 | InChI=1S/C18H26ClNO7/c1-10-8-18(24,11(2)19)16(22)27-13-5-7-20(25)6-4-12(14(13)20)9-26-15(21)17(10,3)23/h4,10-11,13-14,23-24H,5-9H2,1-3H3/t10-,11?,13-,14-,17-,18+,20?/m1/s1 | 1148039-75-1 | Commercial | N/A | 1 | 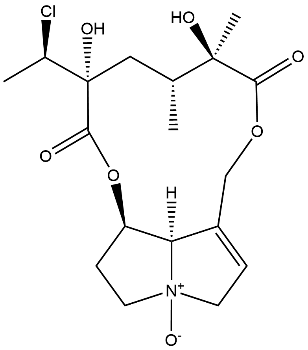 |
| **Junceine** | [M+H]+ | 370.1860 | 8.07 | R | 11MC | C[C@@]1(O)[C@H](C(=O)O[C@@H]2CCN3CC=C(COC(=O)[C@@]1(O)CO)[C@@H]32)C(C)C | InChI=1S/C18H27NO7/c1-10(2)13-15(21)26-12-5-7-19-6-4-11(14(12)19)8-25-16(22)18(24,9-20)17(13,3)23/h4,10,12-14,20,23-24H,5-9H2,1-3H3/t12-,13+,14-,17?,18?/m1/s1 | 480-53-5 | Commercial | N/A | 1 | 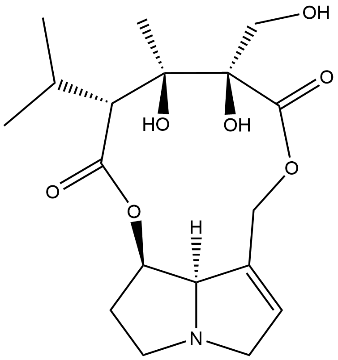 |
| **Junceine N-oxide** | [M+H]+ [2M+H]+ | 386.1810 771.3546 | 4.91 | R | 11MC | C[C@@]1(O)[C@H](C(=O)O[C@@H]2CC[N+]3([O-])CC=C(COC(=O)[C@@]1(O)CO)[C@H]23)C(C)C | InChI=1S/C18H27NO8/c1-10(2)13-15(21)27-12-5-7-19(25)6-4-11(14(12)19)8-26-16(22)18(24,9-20)17(13,3)23/h4,10,12-14,20,23-24H,5-9H2,1-3H3/t12-,13+,14-,17?,18?,19?/m1/s1 | N/A | Commercial | N/A | 1 | 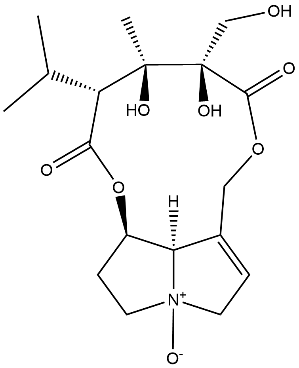 |
| **Lasiocarpine** | [M+H]+ [M+Na]+ | 412.2330 434.2149 | 11.65 | H | DE | CO[C@H](C)C(O)(C(=O)OCC1=CCN2CC[C@H](OC(=O)\C(C)=C/C)[C@H]21)C(C)(C)O | InChI=1S/C21H33NO7/c1-7-13(2)18(23)29-16-9-11-22-10-8-15(17(16)22)12-28-19(24)21(26,14(3)27-6)20(4,5)25/h7-8,14,16-17,25-26H,9-12H2,1-6H3/b13-7-/t14-,16+,17-,21?/m1/s1 | 303-34-4 | Commercial | N/A | 1 | 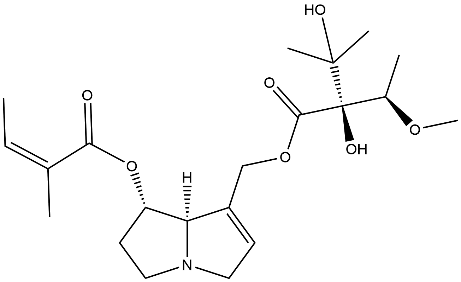 |
| **Lasiocarpine N-oxide** | [M+H]+ [2M+H]+ | 428.2279 855.4485 | 8.84 | H | DE | CO[C@H](C)C(O)(C(=O)OCC1=CC[N+]2([O-])CC[C@H](OC(=O)\C(C)=C/C)[C@@H]12)C(C)(C)O | InChI=1S/C21H33NO8/c1-7-13(2)18(23)30-16-9-11-22(27)10-8-15(17(16)22)12-29-19(24)21(26,14(3)28-6)20(4,5)25/h7-8,14,16-17,25-26H,9-12H2,1-6H3/b13-7-/t14-,16+,17-,21?,22?/m1/s1 | 127-30-0 | Commercial | N/A | 1 | 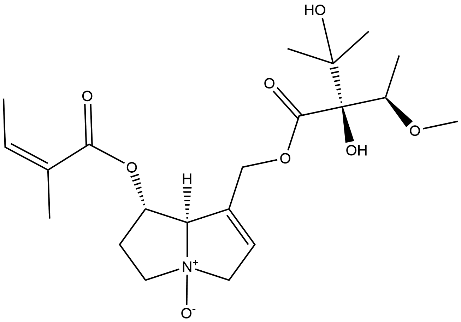 |
| **Lycopsamine** | [M+H]+ | 300.1805 | 5.9 | R | ME | C[C@H](O)[C@@](O)(C(C)C)C(=O)OCC1=CCN2CC[C@@H](O)[C@H]21 | InChI=1S/C15H25NO5/c1-9(2)15(20,10(3)17)14(19)21-8-11-4-6-16-7-5-12(18)13(11)16/h4,9-10,12-13,17-18,20H,5-8H2,1-3H3/t10-,12+,13+,15?/m0/s1 | 10285-07-01 | Commercial | N/A | 1 | 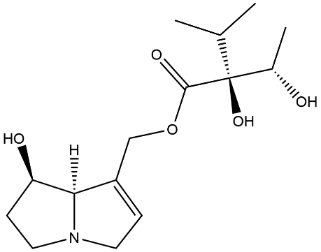 |
| **Lycopsamine N-oxide** | [M+H]+ [2M+H]+ | 316.1755 631.3437 | 3.86 | R | ME | C[C@H](O)[C@@](O)(C(C)C)C(=O)OCC1=CC[N+]2([O-])CC[C@@H](O)[C@@H]12 | InChI=1S/C15H25NO6/c1-9(2)15(20,10(3)17)14(19)22-8-11-4-6-16(21)7-5-12(18)13(11)16/h4,9-10,12-13,17-18,20H,5-8H2,1-3H3/t10-,12+,13+,15?,16?/m0/s1 | 95462-15-0 | Commercial | N/A | 1 | 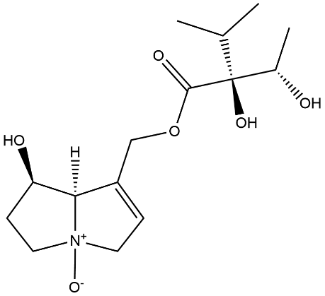 |
| **Merenskine** | [M+H]+ | 388.1521 | 9.75 | R | 12MC | C[C@]1(O)C(=O)OCC2=CCN3CC[C@@H](OC(=O)[C@@](O)(CCl)[C@H](C)[C@H]1C)[C@H]32 | InChI=1S/C18H26ClNO6/c1-10-11(2)18(24,9-19)16(22)26-13-5-7-20-6-4-12(14(13)20)8-25-15(21)17(10,3)23/h4,10-11,13-14,23-24H,5-9H2,1-3H3/t10-,11-,13-,14-,17-,18-/m1/s1 | 96657-94-2 | Commercial | N/A | 1 | 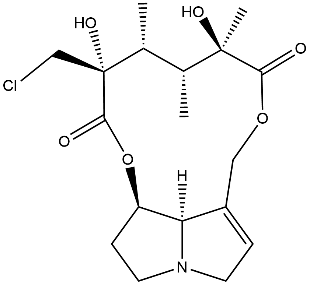 |
| **Merenskine N-oxide** | [M+H]+ [2M+H]+ | 404.1471 807.2868 | 5.61 | R | 12MC | C[C@]1(O)C(=O)OCC2=CC[N+]3([O-])CC[C@@H](OC(=O)[C@@](O)(CCl)[C@H](C)[C@H]1C)[C@@H]23 | InChI=1S/C18H26ClNO7/c1-10-11(2)18(24,9-19)16(22)27-13-5-7-20(25)6-4-12(14(13)20)8-26-15(21)17(10,3)23/h4,10-11,13-14,23-24H,5-9H2,1-3H3/t10-,11-,13-,14-,17-,18-,20?/m1/s1 | 96657-95-3 | Commercial | N/A | 1 | 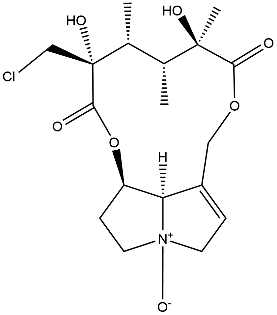 |
| **Merepoxine** | [M+H]+ | 352.1755 | 8.8 | R | 12MC | C[C@]1(O)C(=O)OCC2=CCN3CC[C@@H](OC(=O)[C@@]4(CO4)[C@H](C)[C@H]1C)[C@H]32 | InChI=1S/C18H25NO6/c1-10-11(2)18(9-24-18)16(21)25-13-5-7-19-6-4-12(14(13)19)8-23-15(20)17(10,3)22/h4,10-11,13-14,22H,5-9H2,1-3H3/t10-,11-,13-,14-,17-,18-/m1/s1 | 115777-94-1 | Commercial | N/A | 1 | 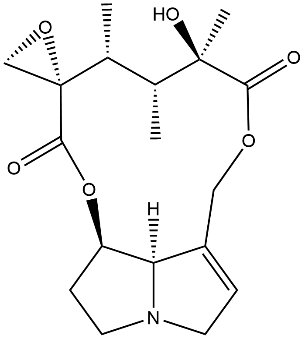 |
| **Merepoxine N-oxide** | [M+H]+ [2M+H]+ | 368.1704 735.3335 | 5.47 | R | 12MC | C[C@]1(O)C(=O)OCC2=CC[N+]3([O-])CC[C@@H](OC(=O)[C@@]4(CO4)[C@H](C)[C@H]1C)[C@@H]23 | InChI=1S/C18H25NO7/c1-10-11(2)18(9-25-18)16(21)26-13-5-7-19(23)6-4-12(14(13)19)8-24-15(20)17(10,3)22/h4,10-11,13-14,22H,5-9H2,1-3H3/t10-,11-,13-,14-,17-,18-,19?/m1/s1 | N/A | Commercial | N/A | 1 | 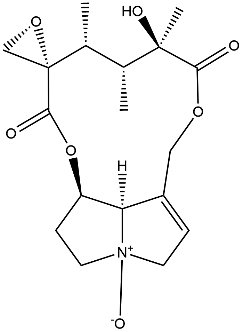 |
| **Monocrotaline** | [M+H]+ | 326.1598 | 6.1 | R | 11MC | C[C@]1(O)C(=O)OCC2=CCN3CC[C@@H](OC(=O)[C@H](C)[C@]1(O)C)[C@H]32 | InChI=1S/C16H23NO6/c1-9-13(18)23-11-5-7-17-6-4-10(12(11)17)8-22-14(19)16(3,21)15(9,2)20/h4,9,11-12,20-21H,5-8H2,1-3H3/t9-,11+,12+,15?,16?/m0/s1 | 315-22-0 | Commercial | N/A | 1 | 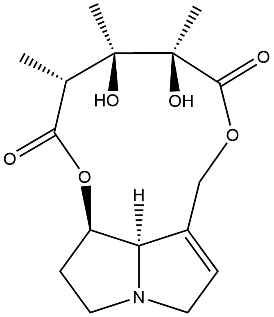 |
| **Monocrotaline N-oxide** | [M+H]+ [2M+H]+ | 342.1547 683.3022 | 11.1 | R | 11MC | C[C@]1(O)C(=O)OCC2=CC[N+]3([O-])CC[C@@H](OC(=O)[C@H](C)[C@]1(O)C)[C@@H]23 | InChI=1S/C16H23NO7/c1-9-13(18)24-11-5-7-17(22)6-4-10(12(11)17)8-23-14(19)16(3,21)15(9,2)20/h4,9,11-12,20-21H,5-8H2,1-3H3/t9-,11+,12+,15?,16?,17?/m0/s1 | 35337-98-5 | Commercial | N/A | 1 | 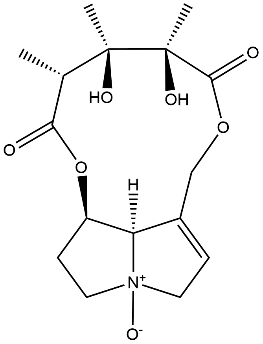 |
| **Monocrotaline-12,13-acetal** | [M+H]+ | 338.1602 | 2.82 | R | 11MC | C[C@]12OCO[C@]2(C)[C@@H](C)C(=O)O[C@@H]2CCN3CC=C(COC1=O)[C@@H]32 | InChI=1S/C17H23NO6/c1-10-14(19)24-12-5-7-18-6-4-11(13(12)18)8-21-15(20)17(3)16(10,2)22-9-23-17/h4,10,12-13H,5-9H2,1-3H3/t10-,12+,13+,16+,17-/m0/s1 | N/A | Commercial | N/A | 2 | 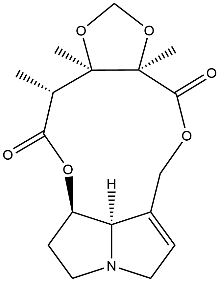 |
| **Otosenine** | [M+H]+ | 382.1860 | 5.05 | O | 12MC | CN1CC[C@H]2OC(=O)[C@@]3(C[C@@H](C)[C@@](C)(O)C(=O)OCC(=CC1)C2=O)O[C@H]3C | InChI=1S/C19H27NO7/c1-11-9-19(12(2)27-19)17(23)26-14-6-8-20(4)7-5-13(15(14)21)10-25-16(22)18(11,3)24/h5,11-12,14,24H,6-10H2,1-4H3/b13-5-/t11-,12+,14-,18-,19+/m1/s1 | 16958-29-5 | Commercial | N/A | 1 | 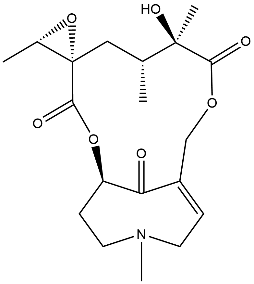 |
| **Platynecine** | [M+H]+ | 158.1176 | 1.53 | P | FB | O[C@@H]1CCN2CC[C@H](CO)[C@@H]21 | InChI=1S/C8H15NO2/c10-5-6-1-3-9-4-2-7(11)8(6)9/h6-8,10-11H,1-5H2/t6-,7-,8-/m1/s1 | 520-62-7 | Isolated | N/A | 1 | 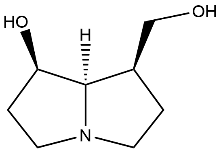 |
| **Platynecine N-oxide** | [M+H]+ [2M+H]+ | 174.1125 347.2177 | 1.29 | P | FB | [O-][N+]12CC[C@@H](O)[C@H]2[C@@H](CO)CC1 | InChI=1S/C8H15NO3/c10-5-6-1-3-9(12)4-2-7(11)8(6)9/h6-8,10-11H,1-5H2/t6-,7-,8-,9?/m1/s1 | N/A | Isolated | N/A | 1 | 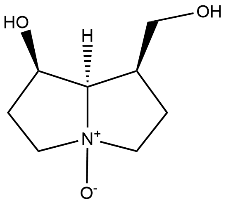 |
| **Platyphylline** | [M+H]+ | 338.1962 | 9.85 | P | 12MC | C[C@]1(O)C(=O)OC[C@H]2CCN3CC[C@@H](OC(=O)\C(C[C@H]1C)=C/C)[C@H]32 | InChI=1S/C18H27NO5/c1-4-12-9-11(2)18(3,22)17(21)23-10-13-5-7-19-8-6-14(15(13)19)24-16(12)20/h4,11,13-15,22H,5-10H2,1-3H3/b12-4-/t11-,13-,14-,15-,18-/m1/s1 | 480-78-4 | Isolated | N/A | 1 | 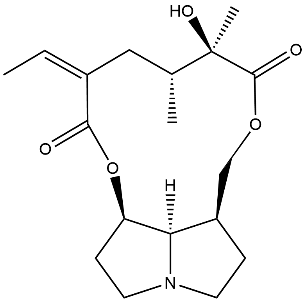 |
| **Platyphylline N-oxide** | [M+H]+ [2M+H]+ | 354.1911 707.3750 | 6.9 | P | 12MC | C[C@]1(O)C(=O)OC[C@H]2CC[N+]3([O-])CC[C@@H](OC(=O)\C(C[C@H]1C)=C/C)[C@@H]23 | InChI=1S/C18H27NO6/c1-4-12-9-11(2)18(3,22)17(21)24-10-13-5-7-19(23)8-6-14(15(13)19)25-16(12)20/h4,11,13-15,22H,5-10H2,1-3H3/b12-4-/t11-,13-,14-,15-,18-,19?/m1/s1 | N/A | Isolated | N/A | 1 | 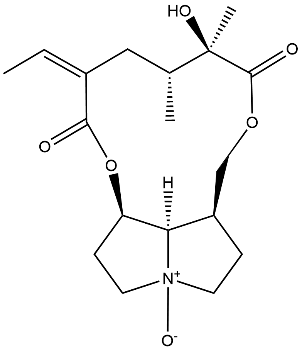 |
| **Retronecine** | [M+H]+ | 156.1019 | 1.77 | R | FB | O[C@@H]1CCN2CC=C(CO)[C@@H]21 | InChI=1S/C8H13NO2/c10-5-6-1-3-9-4-2-7(11)8(6)9/h1,7-8,10-11H,2-5H2/t7-,8-/m1/s1 | 480-85-3 | Commercial | N/A | 1 | 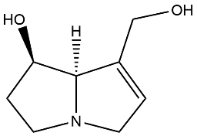 |
| **Retrorsine** | [M+H]+ [M+Na]+ | 352.1755 374.1574 | 9.12 | R | 12MC | O[C@@]1(CO)C(=O)OCC2=CCN3CC[C@@H](OC(=O)\C(C[C@H]1C)=C/C)[C@H]32 | InChI=1S/C18H25NO6/c1-3-12-8-11(2)18(23,10-20)17(22)24-9-13-4-6-19-7-5-14(15(13)19)25-16(12)21/h3-4,11,14-15,20,23H,5-10H2,1-2H3/b12-3-/t11-,14-,15-,18-/m1/s1 | 480-54-6 | Commercial | N/A | 1 | 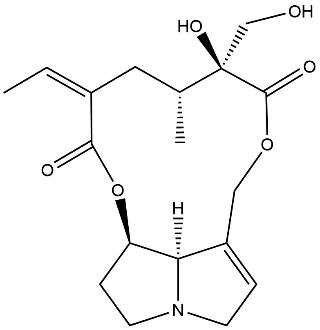 |
| **Retrorsine N-oxide** | [M+H]+ [2M+H]+ | 368.1704 735.3339 | 5.8 | R | 12MC | [O-][N+]12CC[C@@H]3OC(=O)\C(C[C@@H](C)[C@](O)(CO)C(=O)OCC(=CC1)[C@@H]32)=C/C | InChI=1S/C18H25NO7/c1-3-12-8-11(2)18(23,10-20)17(22)25-9-13-4-6-19(24)7-5-14(15(13)19)26-16(12)21/h3-4,11,14-15,20,23H,5-10H2,1-2H3/b12-3-/t11-,14+,15+,18-,19?/m1/s1 | N/A | Commercial | N/A | 1 | 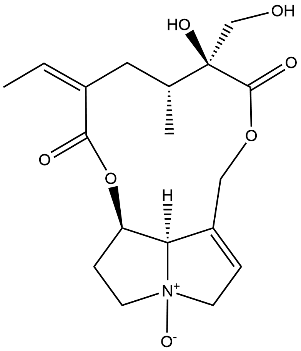 |
| **Riddelliine** | [M+H]+ [M+Na]+ | 350.1598 372.1418 | 8.32 | R | 12MC | O[C@@]1(CO)C(=O)OCC2=CCN3CC[C@@H](OC(=O)\C(CC1=C)=C/C)[C@H]32 | InChI=1S/C18H23NO6/c1-3-12-8-11(2)18(23,10-20)17(22)24-9-13-4-6-19-7-5-14(15(13)19)25-16(12)21/h3-4,14-15,20,23H,2,5-10H2,1H3/b12-3-/t14-,15-,18-/m1/s1 | 23246-96-0 | Commercial | N/A | 1 | 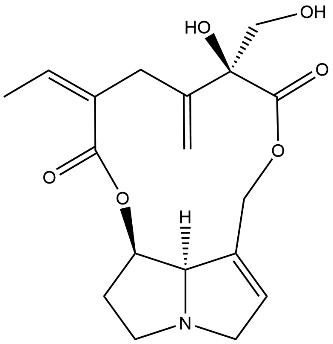 |
| **Riddelliine N-oxide** | [M+H]+ [2M+H]+ | 366.1547 731.3022 | 3.65 | R | 12MC | [O-][N+]12CC[C@H]3OC  (=O)\C(CC(=C)[C@](O)(CO)C(=O)OCC(=CC1)[C@H]32)=C/C | InChI=1S/C18H23NO7/c1-3-12-8-11(2)18(23,10-20)17(22)25-9-13-4-6-19(24)7-5-14(15(13)19)26-16(12)21/h3-4,14-15,20,23H,2,5-10H2,1H3/b12-3-/t14-,15-,18-,19?/m1/s1 | 75056-11-0 | Commercial | N/A | 1 | 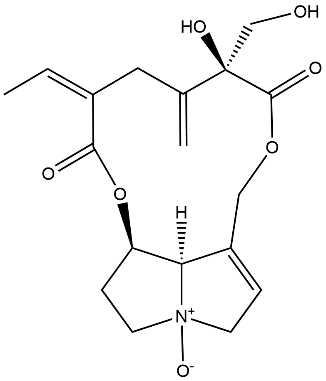 |
| **Rinderine** | [M+H]+ | 300.1805 | 7 | H | ME | C[C@@H](O)[C@@](O)(C(C)C)C(=O)OCC1=CCN2CC[C@H](O)[C@H]21 | InChI=1S/C15H25NO5/c1-9(2)15(20,10(3)17)14(19)21-8-11-4-6-16-7-5-12(18)13(11)16/h4,9-10,12-13,17-18,20H,5-8H2,1-3H3/t10-,12+,13-,15?/m1/s1 | 6029-84-1 | Commercial | N/A | 1 | 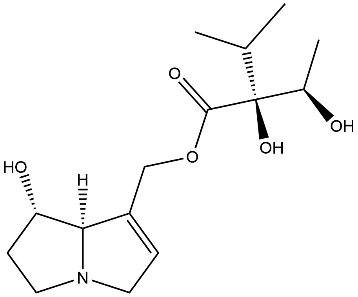 |
| **Rinderine N-oxide** | [M+H]+ [2M+H]+ | 316.1755 631.3437 | 4.29 | H | ME | C[C@@H](O)[C@@](O)(C(C)C)C(=O)OCC1=CC[N+]2([O-])CC[C@H](O)[C@@H]12 | InChI=1S/C15H25NO6/c1-9(2)15(20,10(3)17)14(19)22-8-11-4-6-16(21)7-5-12(18)13(11)16/h4,9-10,12-13,17-18,20H,5-8H2,1-3H3/t10-,12+,13-,15?,16?/m1/s1 | 137821-16-0 | Commercial | N/A | 1 | 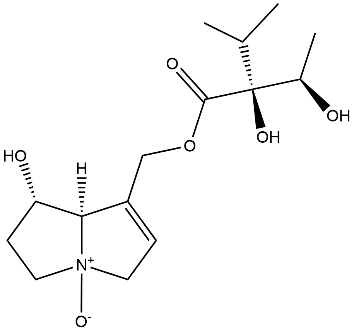 |
| **Rosmarinine** | [M+H]+ | 354.1911 | 8.93 | Ros | 12MC | C[C@]1(O)C(=O)OC[C@@H]2[C@H]3N(C[C@H]2O)CC[C@H]3OC(=O)\C(C[C@H]1C)=C/C | InChI=1S/C18H27NO6/c1-4-11-7-10(2)18(3,23)17(22)24-9-12-13(20)8-19-6-5-14(15(12)19)25-16(11)21/h4,10,12-15,20,23H,5-9H2,1-3H3/b11-4-/t10-,12+,13-,14-,15-,18-/m1/s1 | 520-65-0 | Commercial | N/A | 1 | 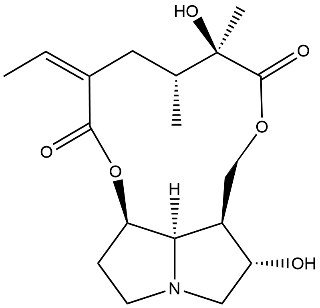 |
| **Rosmarinine N-oxide** | [M+H]+ [2M+H]+ | 370.1860 739.3648 | 6.64 | Ros | 12MC | C[C@]1(O)C(=O)OC[C@@H]2[C@@H]3[C@@H](CC[N+]3([O-])C[C@H]2O)OC(=O)\C(C[C@H]1C)=C/C | InChI=1S/C18H27NO7/c1-4-11-7-10(2)18(3,23)17(22)25-9-12-13(20)8-19(24)6-5-14(15(12)19)26-16(11)21/h4,10,12-15,20,23H,5-9H2,1-3H3/b11-4-/t10-,12+,13-,14-,15-,18-,19?/m1/s1 | 149415-56-5 | Commercial | N/A | 1 | 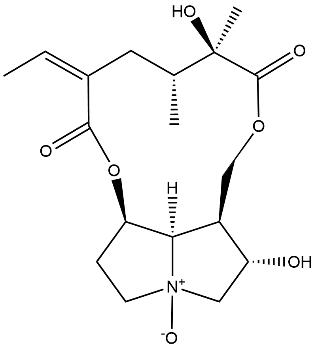 |
| **Sceleratine** | [M+H]+ | 370.1860 | 6.28 | R | 12MC | C[C@]1(O)C(=O)OCC2=CCN3CC[C@@H](OC(=O)[C@@](O)(CO)[C@H](C)[C@H]1C)[C@H]32 | InChI=1S/C18H27NO7/c1-10-11(2)18(24,9-20)16(22)26-13-5-7-19-6-4-12(14(13)19)8-25-15(21)17(10,3)23/h4,10-11,13-14,20,23-24H,5-9H2,1-3H3/t10-,11-,13-,14-,17-,18-/m1/s1 | 6190-26-6 | Commercial | N/A | 1 | 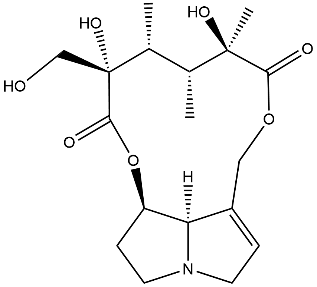 |
| **Sceleratine N-oxide** | [M+H]+ [2M+H]+ | 386.1809 771.3546 | 3.24 | R | 12MC | C[C@]1(O)C(=O)OCC2=CC[N+]3([O-])CC[C@@H](OC(=O)[C@@](O)(CO)[C@H](C)[C@H]1C)[C@@H]23 | InChI=1S/C18H27NO8/c1-10-11(2)18(24,9-20)16(22)27-13-5-7-19(25)6-4-12(14(13)19)8-26-15(21)17(10,3)23/h4,10-11,13-14,20,23-24H,5-9H2,1-3H3/t10-,11-,13-,14-,17-,18-,19?/m1/s1 | 103184-92-5 | Commercial | N/A | 1 | 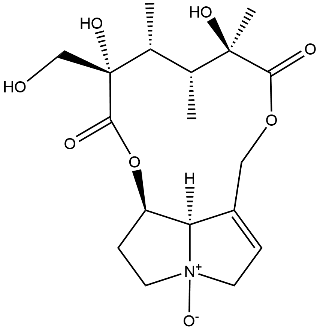 |
| **Senecionine** | [M+H]+ | 336.1805 | 10.8 | R | 12MC | C[C@]1(O)C(=O)OCC2=CCN3CC[C@@H](OC(=O)\C(C[C@H]1C)=C/C)[C@H]32 | InChI=1S/C18H25NO5/c1-4-12-9-11(2)18(3,22)17(21)23-10-13-5-7-19-8-6-14(15(13)19)24-16(12)20/h4-5,11,14-15,22H,6-10H2,1-3H3/b12-4-/t11-,14-,15-,18-/m1/s1 | 130-01-8 | Commercial | N/A | 1 | 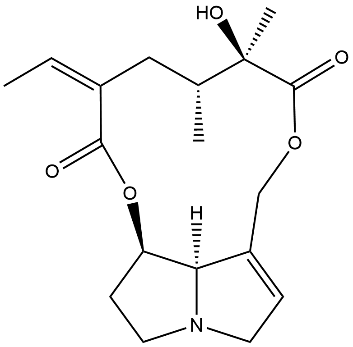 |
| **Senecionine N-oxide** | [M+H]+ [2M+H]+ | 352.1755 703.3437 | 7.19 | R | 12MC | C[C@]1(O)C(=O)OCC2=CC[N+]3([O-])CC[C@@H](OC(=O)\C(C[C@H]1C)=C/C)[C@@H]23 | InChI=1S/C18H25NO6/c1-4-12-9-11(2)18(3,22)17(21)24-10-13-5-7-19(23)8-6-14(15(13)19)25-16(12)20/h4-5,11,14-15,22H,6-10H2,1-3H3/b12-4-/t11-,14-,15-,18-,19?/m1/s1 | 13268-67-2 | Commercial | N/A | 1 | 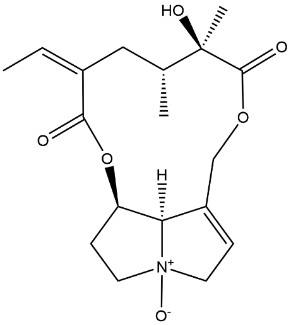 |
| **Seneciphylline** | [M+H]+ | 334.1649 | 9.91 | R | 12MC | C[C@]1(O)C(=O)OCC2=CCN3CC[C@H](OC(=O)\C(CC1=C)=C/C)[C@@H]32 | InChI=1S/C18H23NO5/c1-4-12-9-11(2)18(3,22)17(21)23-10-13-5-7-19-8-6-14(15(13)19)24-16(12)20/h4-5,14-15,22H,2,6-10H2,1,3H3/b12-4-/t14-,15-,18+/m0/s1 | 480-81-9 | Commercial | N/A | 1 | 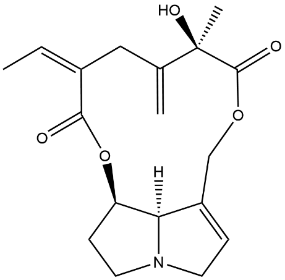 |
| **Seneciphylline N-oxide** | [M+H]+ [2M+H]+ | 350.1598 699.3124 | 6.3 | R | 12MC | C[C@]1(O)C(=O)OCC2=CC[N+]3([O-])CC[C@@H](OC(=O)\C(CC1=C)=C/C)[C@@H]23 | InChI=1S/C18H23NO6/c1-4-12-9-11(2)18(3,22)17(21)24-10-13-5-7-19(23)8-6-14(15(13)19)25-16(12)20/h4-5,14-15,22H,2,6-10H2,1,3H3/b12-4-/t14-,15-,18-,19?/m1/s1 | 38710-26-8 | Commercial | N/A | 1 | 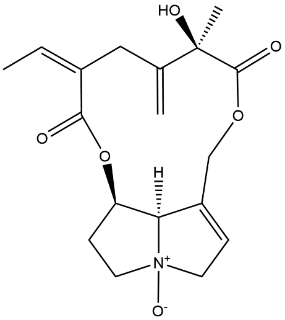 |
| **Senecivernine** | [M+H]+ | 336.1805 | 10.93 | R | 12MC | C[C@]1(O)C(=O)OCC2=CCN3CC[C@@H](OC(=O)C(=C)[C@H](C)[C@H]1C)[C@H]32 | InChI=1S/C18H25NO5/c1-10-11(2)16(20)24-14-6-8-19-7-5-13(15(14)19)9-23-17(21)18(4,22)12(10)3/h5,10,12,14-15,22H,2,6-9H2,1,3-4H3/t10-,12+,14+,15+,18+/m0/s1 | 72755-25-0 | Commercial | N/A | 1 | 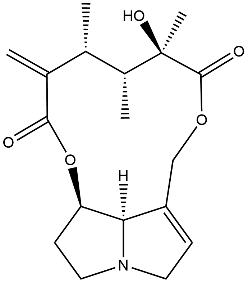 |
| **Senecivernine N-oxide** | [M+H]+ [2M+H]+ | 352.1755 703.3437 | 7.25 | R | 12MC | C[C@]1(O)C(=O)OCC2=CC[N+]3([O-])CC[C@H](OC(=O)C(=C)C(C)[C@H]1C)[C@H]23 | InChI=1S/C18H25NO6/c1-10-11(2)16(20)25-14-6-8-19(23)7-5-13(15(14)19)9-24-17(21)18(4,22)12(10)3/h5,10,12,14-15,22H,2,6-9H2,1,3-4H3/t10?,12-,14+,15+,18-,19?/m1/s1 | 101687-28-9 | Commercial | N/A | 1 | 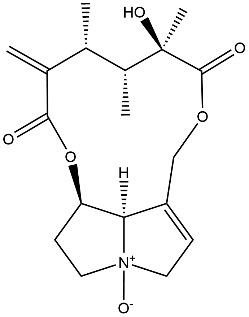 |
| **Senkirkine** | [M+H]+ | 366.1911 | 7.61 | O | 12MC | CN1CC[C@@H]2OC(=O)\C(=C/C)C[C@@H](C)[C@@](C)(O)C(=O)OCC(=CC1)C2=O | InChI=1S/C19H27NO6/c1-5-13-10-12(2)19(3,24)18(23)25-11-14-6-8-20(4)9-7-15(16(14)21)26-17(13)22/h5-6,12,15,24H,7-11H2,1-4H3/b13-5-,14-6-/t12-,15+,19-/m1/s1 | 2318-18-5 | Commercial | N/A | 1 | 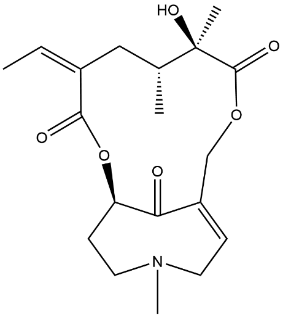 |
| **Spartioidine N-oxide** | [M+H]+ [2M+H]+ | 350.1598 699.3131 | 9.62 | R | 12MC | C[C@]1(O)C(=O)OCC2=CC[N+]3([O-])CC[C@@H](OC(=O)/C(CC1=C)=C/C)[C@@H]23 | InChI=1S/C18H23NO6/c1-4-12-9-11(2)18(3,22)17(21)24-10-13-5-7-19(23)8-6-14(15(13)19)25-16(12)20/h4-5,14-15,22H,2,6-10H2,1,3H3/b12-4+/t14-,15-,18-,19?/m1/s1 | 121123-61-3 | Commercial | N/A | 1 | 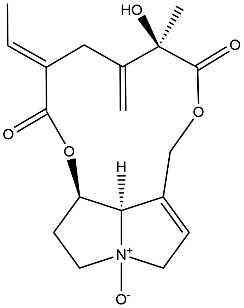 |
| **Spartioidine** | [M+H]+ | 334.1649 | 6.19 | R | 12MC | C[C@]1(O)C(=O)OCC2=CCN3CC[C@@H](OC(=O)/C(CC1=C)=C/C)[C@H]32 | InChI=1S/C18H23NO5/c1-4-12-9-11(2)18(3,22)17(21)23-10-13-5-7-19-8-6-14(15(13)19)24-16(12)20/h4-5,14-15,22H,2,6-10H2,1,3H3/b12-4+/t14-,15-,18-/m1/s1 | 520-59-2 | Commercial | N/A | 1 | 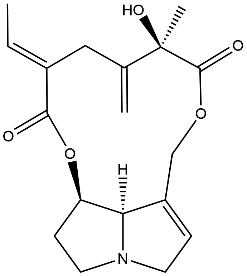 |
| **Thesinine** | [M+H]+ | 288.1594 | 6.03 | T | ME | O=C(\C=C\c1ccc(O)cc1)OC[C@@H]1CCN2CCC[C@@H]21 | InChI=1S/C17H21NO3/c19-15-6-3-13(4-7-15)5-8-17(20)21-12-14-9-11-18-10-1-2-16(14)18/h3-8,14,16,19H,1-2,9-12H2/b8-5+/t14-,16+/m0/s1 | 488-02-8 | Commercial | N/A | 1 | 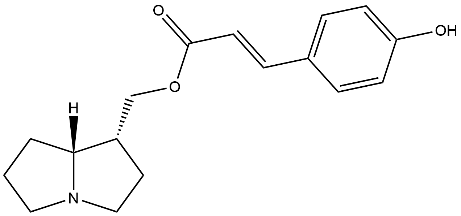 |
| **Thesinine 4'-glucoside** | [M+H]+ | 450.2122 | 7.78 | T | ME | OC1C(OC(CO)C(O)C1O)Oc1ccc(/C=C/C(=O)OC[C@@H]2CCN3CCC[C@@H]32)cc1 | InChI=1S/C23H31NO8/c25-12-18-20(27)21(28)22(29)23(32-18)31-16-6-3-14(4-7-16)5-8-19(26)30-13-15-9-11-24-10-1-2-17(15)24/h3-8,15,17-18,20-23,25,27-29H,1-2,9-13H2/b8-5+/t15-,17+,18?,20?,21?,22?,23?/m0/s1 | 460730-79-4 | Commercial | N/A | 1 | 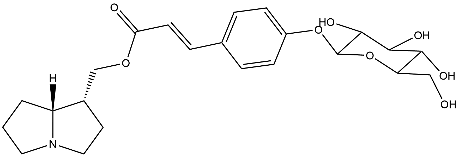 |
| **Trachelanthamine** | [M+H]+ | 286.2013 | 6.36 | T | ME | C[C@@H](O)[C@](O)(C(=O)OC[C@@H]1CCN2CCC[C@H]21)C(C)C | InChI=1S/C15H27NO4/c1-10(2)15(19,11(3)17)14(18)20-9-12-6-8-16-7-4-5-13(12)16/h10-13,17,19H,4-9H2,1-3H3/t11-,12+,13+,15?/m1/s1 | 14140-18-2 | Commercial | N/A | 1 | 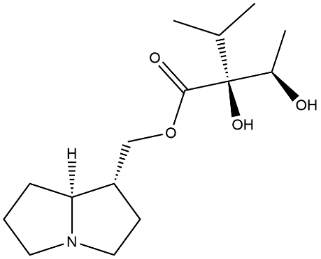 |
| **Trachelanthamine N-oxide** | [M+H]+ [2M+H]+ | 302.1962 603.3851 | 5.59 | T | ME | C[C@@H](O)[C@](O)(C(=O)OC[C@@H]1CC[N+]2([O-])CCC[C@@H]12)C(C)C | InChI=1S/C15H27NO5/c1-10(2)15(19,11(3)17)14(18)21-9-12-6-8-16(20)7-4-5-13(12)16/h10-13,17,19H,4-9H2,1-3H3/t11-,12+,13+,15?,16?/m1/s1 | N/A | Commercial | N/A | 1 | 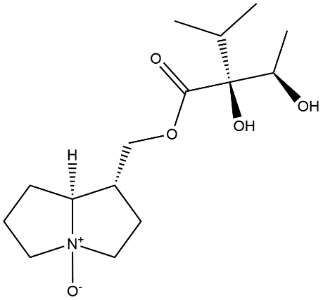 |
| **Trichodesmine** | [M+H]+ | 354.1911 | 9.26 | R | 11MC | C[C@]1(O)C(=O)OCC2=CCN3CC[C@@H](OC(=O)[C@H](C(C)C)[C@@]1(C)O)[C@H]32 | InChI=1S/C18H27NO6/c1-10(2)13-15(20)25-12-6-8-19-7-5-11(14(12)19)9-24-16(21)18(4,23)17(13,3)22/h5,10,12-14,22-23H,6-9H2,1-4H3/t12-,13+,14-,17?,18?/m1/s1 | 548-90-3 | Commercial | N/A | 1 | 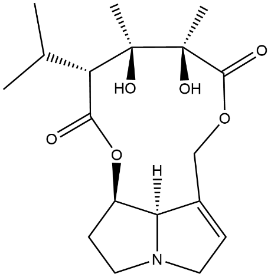 |
| **Trichodesmine N-oxide** | [M+H]+ [2M+H]+ | 370.1860 739.3648 | 5.78 | R | 11MC | C[C@]1(O)C(=O)OCC2=CC[N+]3([O-])CC[C@@H](OC(=O)[C@H](C(C)C)[C@@]1(C)O)[C@@H]23 | InChI=1S/C18H27NO7/c1-10(2)13-15(20)26-12-6-8-19(24)7-5-11(14(12)19)9-25-16(21)18(4,23)17(13,3)22/h5,10,12-14,22-23H,6-9H2,1-4H3/t12-,13+,14-,17?,18?,19?/m1/s1 | 56727-46-3 | Commercial | N/A | 1 | 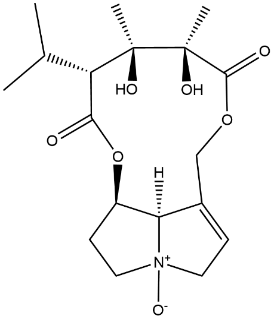 |
| **Usaramine** | [M+H]+ | 352.1755 | 8.82 | R | 12MC | O[C@@]1(CO)C(=O)OCC2=CCN3CC[C@@H](OC(=O)/C(C[C@H]1C)=C/C)[C@H]32 | InChI=1S/C18H25NO6/c1-3-12-8-11(2)18(23,10-20)17(22)24-9-13-4-6-19-7-5-14(15(13)19)25-16(12)21/h3-4,11,14-15,20,23H,5-10H2,1-2H3/b12-3+/t11-,14-,15-,18-/m1/s1 | 15503-87-4 | Commercial | N/A | 1 | 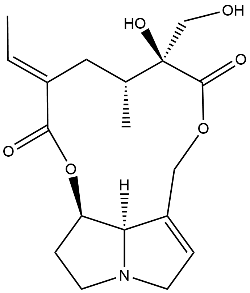 |
| **Usaramine N-oxide** | [M+H]+ [2M+H]+ | 368.1704 735.3335 | 5.69 | R | 12MC | [O-][N+]12CC[C@H]3OC(=O)/C(C[C@@H](C)[C@](O)(CO)C(=O)OCC(=CC1)[C@H]32)=C/C | InChI=1S/C18H25NO7/c1-3-12-8-11(2)18(23,10-20)17(22)25-9-13-4-6-19(24)7-5-14(15(13)19)26-16(12)21/h3-4,11,14-15,20,23H,5-10H2,1-2H3/b12-3+/t11-,14-,15-,18-,19?/m1/s1 | 117020-54-9 | Commercial | N/A | 1 | 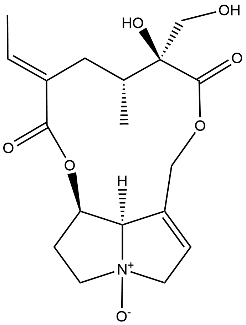 |
| **5'-acetyleuropine** | [M+H]+ [M+Na]+ | 372.2017 394.1836 |  | H | ME | CO[C@H](C)[C@@](O)(C(=O)OCC1=CCN2CC[C@H](O)[C@H]21)C(C)(C)OC(=O)C | InChI=1S/C18H29NO7/c1-11(24-5)18(23,17(3,4)26-12(2)20)16(22)25-10-13-6-8-19-9-7-14(21)15(13)19/h6,11,14-15,21,23H,7-10H2,1-5H3/t11-,14+,15-,18?/m1/s1 | N/A | Crude | Heliotropium europaeum | 3 | 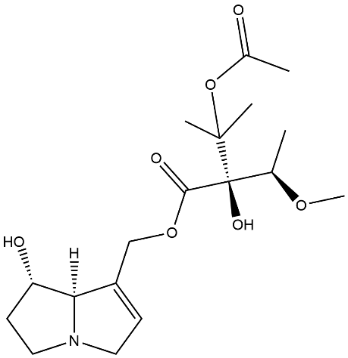 |
| **5'-acetyleuropine N-oxide** | [M+H]+ | 388.1966 |  | H | ME | CO[C@H](C)[C@](O)(C(=O)OCC1=CC[N+]2([O-])CC[C@H](O)[C@@H]12)C(C)(C)OC(=O)C | InChI=1S/C18H29NO8/c1-11(25-5)18(23,17(3,4)27-12(2)20)16(22)26-10-13-6-8-19(24)9-7-14(21)15(13)19/h6,11,14-15,21,23H,7-10H2,1-5H3/t11-,14+,15-,18?,19?/m1/s1 | N/A | Crude | Heliotropium europaeum | 3 | 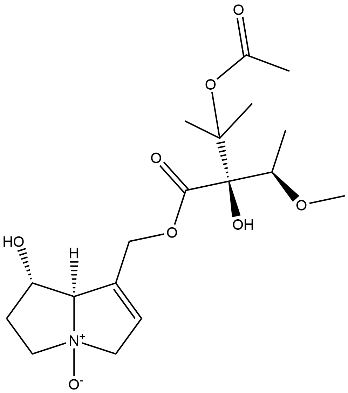 |
| **5'-acetyllasiocarpine** | [M+H]+ [M+Na]+ [2M+H]+ | 454.2435 476.2255 907.4798 |  | H | DE | CO[C@H](C)[C@](O)(C(=O)OCC1=CCN2CC[C@H](OC(=O)\C(C)=C/C)[C@H]21)C(C)(C)OC(C)=O | InChI=1S/C23H35NO8/c1-8-14(2)20(26)31-18-10-12-24-11-9-17(19(18)24)13-30-21(27)23(28,15(3)29-7)22(5,6)32-16(4)25/h8-9,15,18-19,28H,10-13H2,1-7H3/b14-8-/t15-,18+,19-,23?/m1/s1 | N/A | Crude | Heliotropium europaeum | 3 | 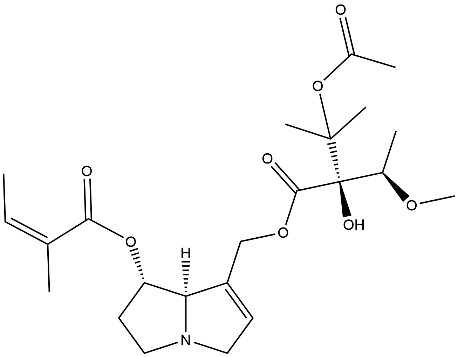 |
| **5'-acetyllasiocarpine N-oxide** | [M+H]+ | 470.2385 |  | H | DE | CO[C@H](C)C(O)(C(=O)OCC1=CC[N+]2([O-])CC[C@H](OC(=O)\C(C)=C/C)[C@@H]12)C(C)(C)OC(C)=O | InChI=1S/C23H35NO9/c1-8-14(2)20(26)32-18-10-12-24(29)11-9-17(19(18)24)13-31-21(27)23(28,15(3)30-7)22(5,6)33-16(4)25/h8-9,15,18-19,28H,10-13H2,1-7H3/b14-8-/t15-,18+,19-,23?,24?/m1/s1 | N/A | Crude | Heliotropium europaeum | 3 | 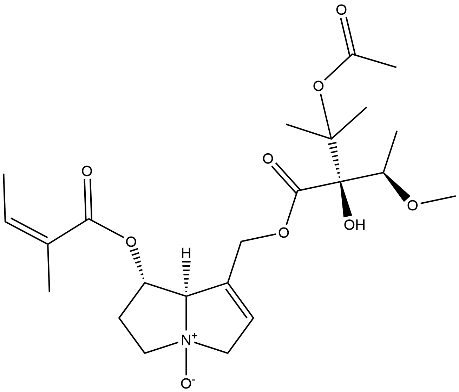 |
| **Acetylerucifoline** | [M+H]+ | 392.1704 | 11.02 | R | 12MC | C[C@]12O[C@]2(C/C(=C/C)C(=O)O[C@@H]2CCN3CC=C(COC1=O)[C@@H]32)COC(=O)C | InChI=1S/C20H25NO7/c1-4-13-9-20(11-26-12(2)22)19(3,28-20)18(24)25-10-14-5-7-21-8-6-15(16(14)21)27-17(13)23/h4-5,15-16H,6-11H2,1-3H3/b13-4-/t15-,16-,19+,20-/m1/s1 | N/A | Crude | Jacobaea vulgaris | 3 | 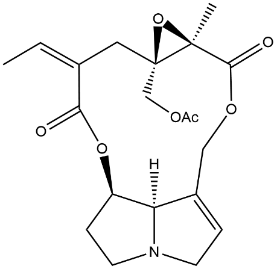 |
| **Acetylerucifoline N-oxide** | [M+H]+ [2M+H]+ | 408.1656 815.3232 | 7.39 | R | 12MC | C[C@]12O[C@]2(C/C(=C/C)C(=O)O[C@@H]2CC[N+]3([O-])CC=C(COC1=O)[C@H]23)COC(=O)C | InChI=1S/C20H25NO8/c1-4-13-9-20(11-27-12(2)22)19(3,29-20)18(24)26-10-14-5-7-21(25)8-6-15(16(14)21)28-17(13)23/h4-5,15-16H,6-11H2,1-3H3/b13-4-/t15-,16-,19+,20-,21?/m1/s1 | N/A | Crude | Jacobaea vulgaris | 3 | 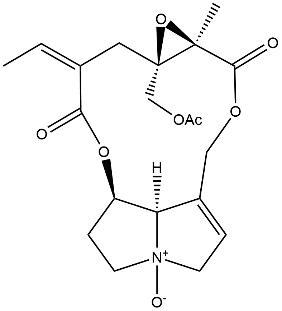 |
| **Acetylseneciphylline** | [M+H]+ | 376.1759 | 11.85 | R | 12MC | C[C@]1(OC(C)=O)C(=O)OCC2=CCN3CC[C@@H](OC(=O)\C(=C/C)CC1=C)[C@H]32 | InChI=1S/C20H25NO6/c1-5-14-10-12(2)20(4,27-13(3)22)19(24)25-11-15-6-8-21-9-7-16(17(15)21)26-18(14)23/h5-6,16-17H,2,7-11H2,1,3-4H3/b14-5-/t16-,17-,20-/m1/s1 | N/A | Crude | Jacobaea arnautorum | 3 | 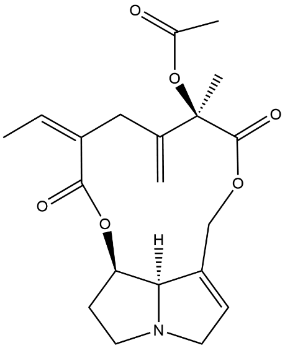 |
| **Acetylseneciphylline N-oxide** | [M+H]+ [M+Na]+ [2M+H]+ | 392.1705 414.1524 783.3333 | 9.72 | R | 12MC | C[C@]1(OC(C)=O)C(=O)OCC2=CC[N+]3([O-])CC[C@@H](OC(=O)\C(=C/C)CC1=C)[C@@H]23 | InChI=1S/C20H25NO7/c1-5-14-10-12(2)20(4,28-13(3)22)19(24)26-11-15-6-8-21(25)9-7-16(17(15)21)27-18(14)23/h5-6,16-17H,2,7-11H2,1,3-4H3/b14-5-/t16-,17-,20-,21?/m1/s1 | N/A | Crude | Jacobaea carniolia | 3 | 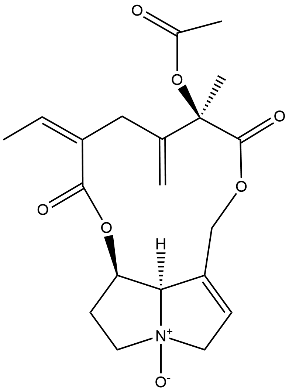 |
| **Acetylsenkirkine** | [M+H]+ [M+Na]+ | 408.2021 430.1837 | 10.98 | O | 12MC | CN1CC=C2COC(=O)[C@](C)(OC(C)=O)[C@H](C)C/C(=C/C)C(=O)O[C@@H](CC1)C2=O | InChI=1S/C21H29NO7/c1-6-15-11-13(2)21(4,29-14(3)23)20(26)27-12-16-7-9-22(5)10-8-17(18(16)24)28-19(15)25/h6-7,13,17H,8-12H2,1-5H3/b15-6-,16-7-/t13-,17+,21-/m1/s1 | N/A | Crude | Jacobaea gnaphalioides | 3 | 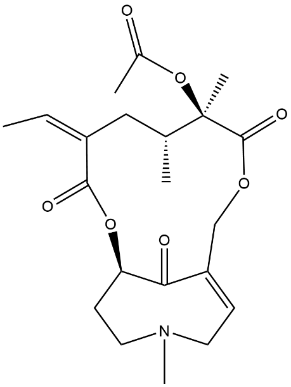 |
| **Adonifoline** | [M+H]+ | 366.1548 | 6.07 | R | 12MC | OC12CC3(COC2C)OC3(C)C(=O)OCC2=CCN3CC[C@@H](OC1=O)[C@H]32 | InChI=1S/C18H23NO7/c1-10-18(22)8-17(9-24-10)16(2,26-17)14(20)23-7-11-3-5-19-6-4-12(13(11)19)25-15(18)21/h3,10,12-13,22H,4-9H2,1-2H3/t10?,12-,13-,16?,17?,18?/m1/s1 | N/A | Crude | Jacobaea adonifolia | 3 | 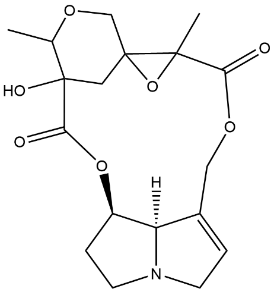 |
| **Adonifoline N-oxide** | [M+H]+ [2M+H]+ | 382.1500 763.2921 | 3.81 | R | 12MC | [O-][N+]12CC[C@H]3OC(=O)C4(O)CC5(COC4C)OC5(C)C(=O)OCC(=CC1)[C@H]32 | InChI=1S/C18H23NO8/c1-10-18(22)8-17(9-25-10)16(2,27-17)14(20)24-7-11-3-5-19(23)6-4-12(13(11)19)26-15(18)21/h3,10,12-13,22H,4-9H2,1-2H3/t10?,12-,13-,16?,17?,18?,19?/m1/s1 | N/A | Crude | Jacobaea adonifolia | 3 | 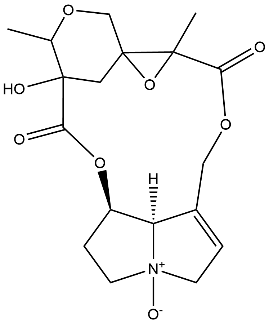 |
| **Desacetyldoronine** | [M+H]+ | 418.1634 | 6.13 | O | 12MC | CN1CC[C@H]2OC(=O)[C@](O)(C[C@@H](C)[C@@](C)(O)C(=O)OCC(=CC1)C2=O)C(Cl)C | InChI=1S/C19H28ClNO7/c1-11-9-19(26,12(2)20)17(24)28-14-6-8-21(4)7-5-13(15(14)22)10-27-16(23)18(11,3)25/h5,11-12,14,25-26H,6-10H2,1-4H3/b13-5-/t11-,12?,14-,18-,19+/m1/s1 | N/A | Crude | Jacobaea aquatica | 3 | 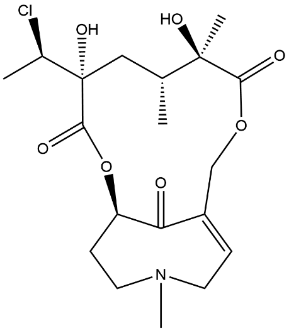 |
| **Dehydrosenkirkine** | [M+H]+ | 364.1760 | 6.84 | O | 12MC | CN1CC[C@H]2OC(=O)\C(=C/C)CC(=C)[C@@](C)(O)C(=O)OCC(=CC1)C2=O | InChI=1S/C19H25NO6/c1-5-13-10-12(2)19(3,24)18(23)25-11-14-6-8-20(4)9-7-15(16(14)21)26-17(13)22/h5-6,15,24H,2,7-11H2,1,3-4H3/b13-5-,14-6-/t15-,19-/m1/s1 | N/A | Crude | Jacobaea aquatica | 3 | 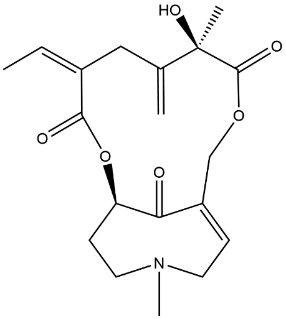 |
| **Doronine** | [M+H]+ | 460.1738 | 9.92 | O | 12MC | CN1CC[C@H]2OC(=O)[C@](O)(C[C@@H](C)[C@@](C)(OC(C)=O)C(=O)OCC(=CC1)C2=O)C(Cl)C | InChI=1S/C21H30ClNO8/c1-12-10-21(28,13(2)22)19(27)30-16-7-9-23(5)8-6-15(17(16)25)11-29-18(26)20(12,4)31-14(3)24/h6,12-13,16,28H,7-11H2,1-5H3/b15-6-/t12-,13?,16-,20-,21+/m1/s1 | N/A | Crude | Jacobaea aquatica | 3 | 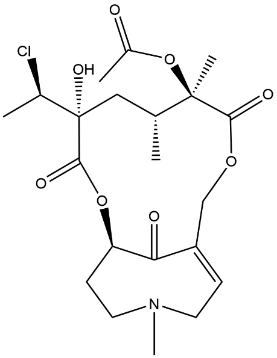 |
| **Floridanine** | [M+H]+ [M+Na]+ | 442.2075 464.1888 | 6.94 | O | 12MC | CN1CC[C@H]2OC(=O)[C@](O)(C[C@@H](C)[C@@](C)(OC(C)=O)C(=O)OCC(=CC1)C2=O)C(O)C | InChI=1S/C21H31NO9/c1-12-10-21(28,13(2)23)19(27)30-16-7-9-22(5)8-6-15(17(16)25)11-29-18(26)20(12,4)31-14(3)24/h6,12-13,16,23,28H,7-11H2,1-5H3/b15-6-/t12-,13?,16-,20-,21+/m1/s1 | N/A | Crude | Jacobaea aquatica | 3 | 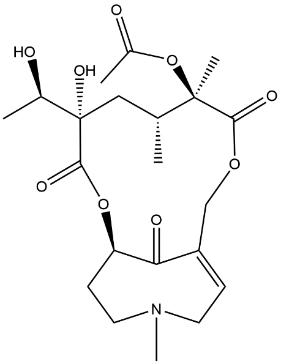 |
| **Florosenine** | [M+H]+ [M+Na]+ | 424.1971 446.1786 | 8.99 | O | 12MC | CN1CC[C@H]2OC(=O)[C@]3(O[C@H]3C)C[C@@H](C)[C@@](C)(OC(C)=O)C(=O)OCC(=CC1)C2=O | InChI=1S/C21H29NO8/c1-12-10-21(13(2)29-21)19(26)28-16-7-9-22(5)8-6-15(17(16)24)11-27-18(25)20(12,4)30-14(3)23/h6,12-13,16H,7-11H2,1-5H3/b15-6-/t12-,13+,16-,20-,21+/m1/s1 | N/A | Crude | Jacobaea alpina | 3 | 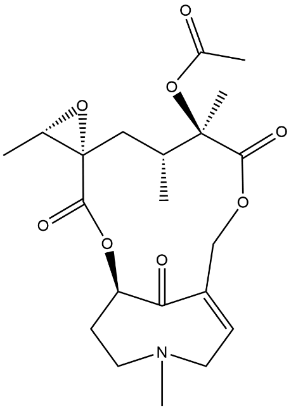 |
| **Heleurine** | [M+H]+ | 298.2013 |  | T | ME | CO[C@H](C)[C@@](O)(C(=O)OCC1=CCN2CCC[C@H]21)C(C)C | InChI=1S/C16H27NO4/c1-11(2)16(19,12(3)20-4)15(18)21-10-13-7-9-17-8-5-6-14(13)17/h7,11-12,14,19H,5-6,8-10H2,1-4H3/t12-,14+,16?/m1/s1 | N/A | Crude | Heliotropium europaeum | 3 | 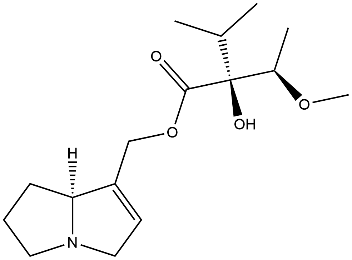 |
| **Heleurine N-oxide** | [M+H]+ [2M+H]+ | 314.1962 628.3840 |  | T | ME | CO[C@H](C)[C@](O)(C(=O)OCC1=CC[N+]2([O-])CCC[C@@H]12)C(C)C | InChI=1S/C16H27NO5/c1-11(2)16(19,12(3)21-4)15(18)22-10-13-7-9-17(20)8-5-6-14(13)17/h7,11-12,14,19H,5-6,8-10H2,1-4H3/t12-,14+,16?,17?/m1/s1 | N/A | Crude | Heliotropium europaeum | 3 | 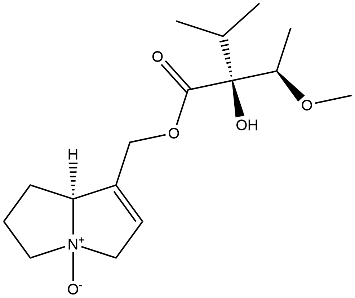 |
| **Jacozine** | [M+H]+ | 350.1602 | 7.39 | R | 12MC | C[C@]1(O)C(=O)OCC2=CCN3CC[C@@H](OC(=O)[C@@]4(CC1=C)O[C@@H]4C)[C@H]32 | InChI=1S/C18H23NO6/c1-10-8-18(11(2)25-18)16(21)24-13-5-7-19-6-4-12(14(13)19)9-23-15(20)17(10,3)22/h4,11,13-14,22H,1,5-9H2,2-3H3/t11?,13-,14-,17-,18+/m1/s1 | N/A | Crude | Jacobaea vulgaris | 3 | 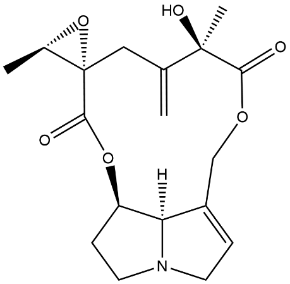 |
| **Jacozine N-oxide** | [M+H]+ [2M+H]+ | 366.1543 731.3034 | 3.76 | R | 12MC | C[C@]1(O)C(=O)OCC2=CC[N+]3([O-])CC[C@@H](OC(=O)[C@@]4(CC1=C)O[C@H]4C)[C@@H]23 | InChI=1S/C18H23NO7/c1-10-8-18(11(2)26-18)16(21)25-13-5-7-19(23)6-4-12(14(13)19)9-24-15(20)17(10,3)22/h4,11,13-14,22H,1,5-9H2,2-3H3/t11-,13+,14+,17+,18-,19?/m0/s1 | N/A | Crude | Jacobaea vulgaris | 3 | 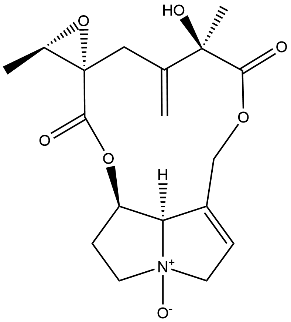 |
| **Neosenkirkine** | [M+H]+ | 366.1915 | 7.41 | O | 12MC | CN1CC[C@H]2OC(=O)/C(=C/C)C[C@@H](C)[C@@](C)(O)C(=O)OCC(=CC1)C2=O | InChI=1S/C19H27NO6/c1-5-13-10-12(2)19(3,24)18(23)25-11-14-6-8-20(4)9-7-15(16(14)21)26-17(13)22/h5-6,12,15,24H,7-11H2,1-4H3/b13-5+,14-6-/t12-,15-,19-/m1/s1 | N/A | Crude | Jacobaea uniflora | 3 |  |
| **Onetine** | [M+H]+ | 400.1970 | 2.86 | O | 12MC | CN1CC[C@H]2OC(=O)[C@](O)(C[C@@H](C)[C@@](C)(O)C(=O)OCC(=CC1)C2=O)[C@H](O)C | InChI=1S/C19H29NO8/c1-11-9-19(26,12(2)21)17(24)28-14-6-8-20(4)7-5-13(15(14)22)10-27-16(23)18(11,3)25/h5,11-12,14,21,25-26H,6-10H2,1-4H3/b13-5-/t11-,12-,14-,18-,19+/m1/s1 | N/A | Crude | Jacobaea aquatica | 3 |  |

## S3: settings Proteowizard MSconvert

## S4: Classic molecular network with labels (Validation step 1)

## S5: Settings mzMine and GNPS for Validation step 3 (FBMN of *Jacobaea gnaphalioides* and *Heliotropium europaeum*)

MzMine3:

- Processing wizard, standard settings for UHPLC-Orbitrap-DDA with the following exceptions:
  - UHPLC:
    - Crop retention time: 0.30-20.00
  - Orbitrap:
    - Noise threshold MS1: 5.0
    - Noise threshold MS2..MSn: 2.0
    - Minimum feature height: 5.0E7
  - Created Batch:
    - Duplicate peak filter: 0.4 min RT tolerance

GNPS:

- FEATURE-BASED-MOLECULAR-NETWORKING (version release_28.2)
  - Basic Network Options:
    - Quantification Table Source: MZmine
    - Precursor Ion Mass Tolerance: 0.02 Da
    - Fragment Ion Mass Tolerance: 0.02 Da
  - Advances Network Options:
    - Min Pairs Cos: 0.8
    - Network TopK: 10
    - Minimum Matched Fragment Ions: 6
  - Advanced Library Search Options:
    - Library Search Min Matched Peaks: 6
    - Score Threshold: 0.7

## S6: FBMN *Heliotropium europaeum* with less strict conditions (minimum feature height 1E6):

## S7: Comparison of PAs annotated in *Jacobaea gnaphalioides* by PAsDB to Chen et al (PAs content above μg/g DW):

| PA Chen et al. | Content [μg/g DW] | PA annotated PAsDB |
| --- | --- | --- |
| Sk (366) | 1071.93 | Senkirkine |
| Pt (338) | 744.08 | Platyphylline |
| AcSp-ox (392) | 659.00 | Acetyl-seneciphylline N-ox |
| Sn-ox (352) | 648.32 | Senecionine N-oxide |
| AcSk (408) | 621.15 | Acetylsenkirkine |
| Sp-ox (350) | 572.30 | Seneciphylline N-oxide |
| Us-ox (368) | 363.92 | Usaramine N-oxide |
| Rt-ox (368) | 317.64 | Retrorsine N-oxide |
| Ir-ox (352) | 251.49 | Integerrimine N-oxide |
| DHSk (364) | 103.01 | Dehydrosenkirkine |
| Sp (334) | 84.18 | Seneciphylline |
| Pt-ox 354 (6.95) | 70.78 | Platyphylline N-oxide |
| Sn (336) | 70.73 | Senecionine |
| Jz (350) | 49.19 | - |
| Jb-ox (368) | 35.76 | Jacobine N-oxide |
| AcSp (376) | 35.27 | Acetyl-seneciphylline |
| Jb (352) | 23.35 | Jacobine |
| Ir (336) | 13.34 | - |
| Rt (352) | 10.44 | Retrorsine |

## S8: FBMN *Jacobaea gnaphalioides* with less strict conditions (minimum feature height 1E6):
